# Diffusion in deforming semi-permeable media: a Monte Carlo random walk approach

**DOI:** 10.1038/s41598-026-64054-1

**Published:** 2026-08-07

**Authors:** Ignasi Alemany, Andrew D. Scott, Denis J. Doorly

**Affiliations:** 1 Department of Aeronautics, Imperial College London, London, UK; 2 Cardiovascular Magnetic Resonance Unit, Royal Brompton Hospital, Guy’s and St Thomas’ NHS Foundation Trust, London, UK; 3 National Heart and Lung Institute, Imperial College London, London, UK

**Keywords:** Biophysics, Engineering, Materials science, Mathematics and computing, Physics

## Abstract

Diffusion processes in deforming media with semi-permeable barriers are fundamental to understanding many biological and physical systems. The presence of semi-permeable barriers causes temporal and spatial departures from normal diffusion. We present an analytic expression for diffusion in a dynamically strained cuboidal cell that demonstrates one extreme of behaviour. Unfortunately however, analytical models cannot represent the effects of dynamic strain in microscopically restrictive media with varying permeability. To address these limitations, we explore how different cyclical strain patterns affect the apparent diffusion tensor in a simplified microstructure inspired by cardiac tissue. We show how the diffusion tensor in a dynamically strained medium may be derived for a Monte Carlo Random Walk (MCRW) model. The MCRW procedure is readily adaptable to more complex geometries and to cyclic and non-cyclic strains. Computations using the MCRW method show that the effects of physiologically relevant strain ($\sim 20\%$) are significantly attenuated by semi-permeable barriers. Strain correction methods, derived for homogeneous diffusion, are found to systematically overcorrect as barrier permeability decreases. These findings are significant for interpreting measurements, particularly in biology, where both microstructural barriers and dynamic deformation alter observed diffusion properties.

## Introduction

Diffusion processes are fundamental to a wide range of physical, biological, and chemical phenomena, describing the transport dynamics of a property concentration or density from regions of high to low concentrations^1^. In physics, diffusion processes include heat conduction^1,2^, particle transport, and charge carrier dynamics in semiconductors^3^; in biology, molecular self-diffusion occurs in transport across cellular membranes^4–6^ at the microscale, whilst diffusion processes model the propagation of diseases through populations at the large scale; in financial mathematics^7^, diffusion underpins stochastic models of price evolution in derivative markets. In complex media, diffusion is often highly restricted and influenced by the structural geometry, barriers and the dynamic deformation of the media^4–6,8,9^. Understanding the influence of these factors is crucial for interpreting diffusion measurements in deformable materials such as biological tissues^10–13^, composite materials^14^, porous materials^15–17^ or soft materials like hydrogels.^18^

If the distribution of some (scalar) quantity *c* (e.g. temperature, mass concentration, particle density etc) spreads in (a classical) diffusive manner, Fick’s first law states that the diffusive flux of *c*, denoted $\boldsymbol {j}$, is given by^19,20^:

1 $$\begin{aligned} \boldsymbol {j} = -\textbf{D}(\boldsymbol {y})\nabla c, \end{aligned}$$

where $\textbf{D}$ is a symmetric tensor that captures directional dependencies^21,22^. Diffusion can be uniform in space (isotropic, $\textbf{D} = D_0\textbf{I}$) or dependent on spatial location $\boldsymbol {y}$ (anisotropic). If the local concentration on one side of a permeable barrier, say $c_1$ differs from that on the other side, $c_2$, there is a diffusive flux across the membrane driven by the concentration difference,

2 $$\begin{aligned} \boldsymbol {n}\cdot \boldsymbol {j}= j_n = \kappa (c_1-c_2) \end{aligned}$$

where $\boldsymbol {n}$ is the normal to the boundary that points from region 1 to region 2, *κ* is the permeability of the boundary (here in units ${\mathrm{\upmu }}\textrm{m}\, \textrm{ms}^{-1}$). According to Fick’s second law, the rate of change of $c(\boldsymbol {y},t)$ with time *t* is given by:

3 $$\begin{aligned} \frac{\partial c(\boldsymbol {y},t)}{\partial t} = -\nabla \cdot \boldsymbol {j} = \nabla \cdot (\textbf{D}(\boldsymbol {y})\nabla c) \end{aligned}$$

Cardiac diffusion tensor imaging (cDTI) provides a means to measure the components of the tensor $\textbf{D}$, that is assumed to describe the Brownian motion of water molecules diffusing in an imaged voxel of tissue. Based on the assumption of an anisotropic, locally homogeneous region, the (6 independent) components of the diffusion tensor $D_{ij}$ in an imaging voxel are derived via the covariances of displacements as $\langle \Delta r_i \Delta r_j \rangle = 2 D_{ij} T$, where $\Delta r_i = r_i(t) - r_i(0)$ represents displacement along axis $i \in \{x,y,z\}$ from the initial position over time *T*. So

4 $$\begin{aligned} \textbf{D} = \frac{1}{2T} \begin{pmatrix} \langle \Delta x^2 \rangle & \langle \Delta x \Delta y \rangle & \langle \Delta x \Delta z \rangle \\ \langle \Delta y \Delta x \rangle & \langle \Delta y^2 \rangle & \langle \Delta y \Delta z \rangle \\ \langle \Delta z \Delta x \rangle & \langle \Delta z \Delta y \rangle & \langle \Delta z^2 \rangle \end{pmatrix} \end{aligned}$$

Whilst the actual process of diffusion in a complex tissue microstructure is not in reality a Gaussian process governed by tensor Eq. (4), fitting the acquired signal data to such a model allows in-vivo cDTI sequences to offer a unique and non-invasive methodology to provide insights into the tissue microstructure^23^, via the apparent diffusion tensor.

In the forward problem, we seek to determine the effects on measurements of $\textbf{D}$ by simulating diffusion with varying microstructure parameters. However whereas Eq. (3) can be solved analytically for simple domains using classical techniques^1,24^, for more complex media where geometry and boundary conditions are intricate, numerical methods become essential. Standard discrete methods, including finite difference (FD)^25^, finite volume (FV)^26^, and finite element methods (FEM)^27^, are commonly employed, but have limited scalability, as they require accurate volumetric meshing and the computational effort to solve in 3D increases substantially. The problem of fitting a volume mesh to match cell boundaries can be alleviated using immersed boundary (IB) methods, which are attractive for deforming boundaries^28^. Another alternative is the Lattice Boltzmann Method (LBM), for example Naughton et al.^29^. The LBM is computationally highly efficient through the use of a regular grid with appropriate collision models to update the solution rather than lengthier discrete approximations to the continuous partial differential equations. The underlying grid in both IB and LBM solvers must be sufficiently fine to model transport across membranes. When dealing with volumes comprising a large range of scales, such as biological tissues containing intricate membrane geometries, where intercellular distances are on the order of microns, $\mathcal {O}({\mathrm{{\upmu }m}})$, this implies a dense grid. Faced with the potential computational cost of grid-based approaches, Monte Carlo random walk (MCRW) simulations^5,30–34^ offer an alternative. MCRW simulations can readily accommodate complex geometries, heterogeneous properties, and dynamic boundary conditions. MCRW simulations are also notably efficient in higher dimensions, and offer simpler handling of interface boundary conditions, such as permeability or reflections. By simulating the trajectories of individual particles that undergo random motion, MCRW methods naturally model the real physical process and are ideally suited to parallel processing.

When the medium itself undergoes deformation, the relationship between the apparent diffusive displacements (as measured in the laboratory) and the underlying structural properties becomes more complex. Analytical models exist for the effects of strain on diffusion in homogeneous media^35^. However biological tissues, in particular cardiac muscle tissue, present a non-homogeneous, dynamic, mesoscopic structure, with varying diffusivity and semi-permeable membranes introducing significant complications; there is moreover contractile activity throughout the cardiac cycle^36^. This intricate and dynamic medium poses significant challenges for investigating water molecule diffusion in cardiac tissue^37^. During contraction and relaxation cycles, cardiomyocytes undergo substantial deformation, with strain values reaching up to $\pm 20\%$ along the principal axes^38^. MR diffusion tensor measurements (e.g. in cDTI) are thus inevitably affected by the medium deformation^10,39,40^, confounding the interpretation of tissue microstructure from diffusion data^13,35^.

Previous approaches to account for strain effects in diffusion measurements have relied on correction methods derived for homogeneous diffusion,^35,41^. These corrections assume the probability distribution function (pdf) for a diffusing particle conforms to a (possibly anisotropic) Gaussian or Normal distribution that can be characterised by a simple covariance matrix. However, the applicability of these methods to biological tissues with semi-permeable membranes and restricted diffusion remains questionable. In this study, we address this gap in our knowledge by implementing a Monte Carlo random walk (MCRW) algorithm to simulate diffusion in deforming media with semi-permeable barriers.

Here we focus on a simplified domain inspired by cardiac tissue, that is easily replicated and that affords some insight through analysis. We consider a broad range of barrier permeabilities to facilitate clear interpretation of strain effects and to provide general results that can be adaptable to other biological tissues or applications. Also to highlight purely the results of strain, we assume the deformation to be incompressible and that the intrinsic diffusion is unaltered. Note however that since the random walk method employed is effectively decoupled from the cell geometry update (see methods) it would be quite straightforward to adapt the methodology presented here to incorporate both compressibility and deformation induced dynamic alterations in intra- and extra-cellular geometry and diffusivity as well as in permeability.

## Results

**Fig. 1:**
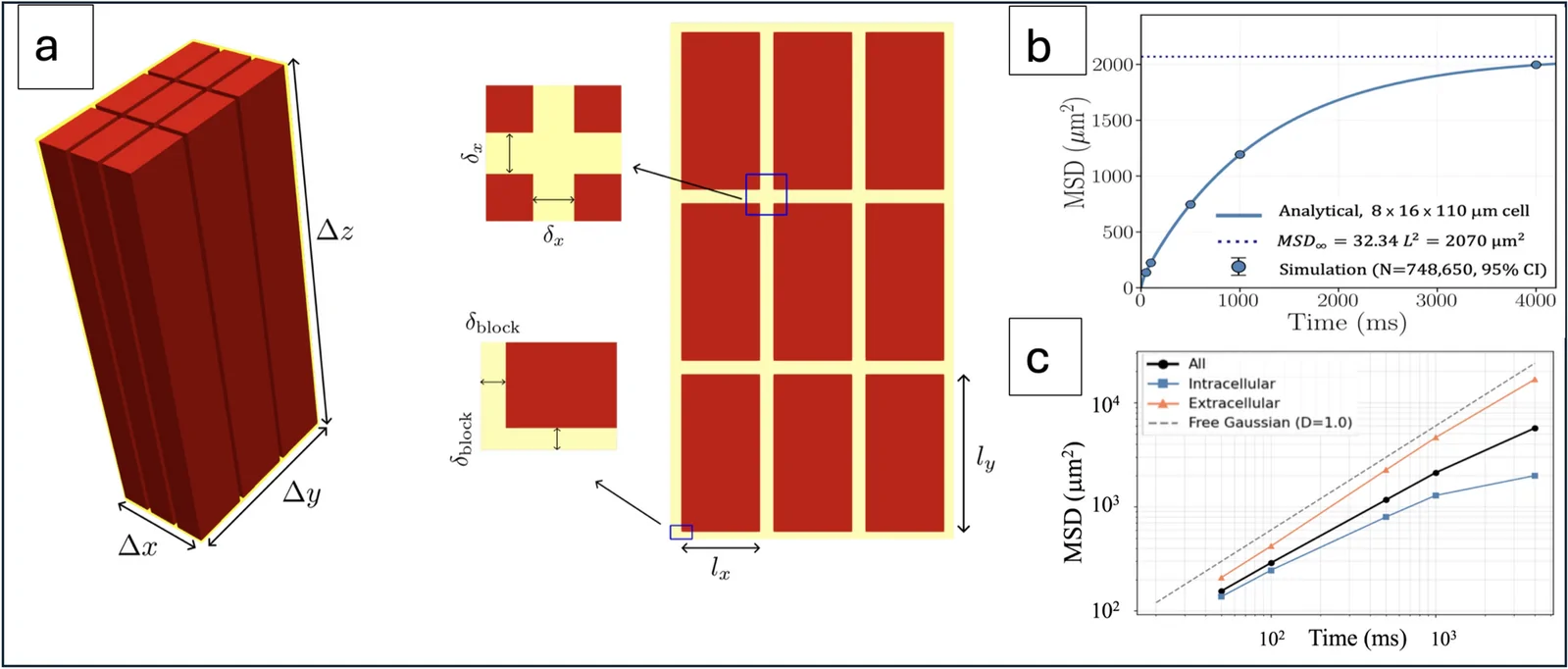
(**a**) Reference geometric model of cardiomyocyte arrangement (C1). Left: 3D representation of the *3× 3* elongated cell array with dimensions $l_x = {8}\mathrm{{\upmu }m}$, $l_y = {16}\mathrm{{\upmu }m}$, and $l_z = {110}\mathrm{{\upmu }m}$. Right: Cross-sectional short-axes (*x*-*y*) view showing intercellular spacing $\delta _x ={1.33}\mathrm{{\upmu }m}$ and boundary spacing $\delta _\text {block} = {1}\mathrm{{\upmu }m}$. The block maintains an extracellular volume fraction (ECV) of 25%, consistent with healthy cardiac tissue and is repeated periodically to fill a cubic voxel 2.8*×*2.8*×*2.8 mm$^3$. (**b**) Evolution of calculated intra-cellular mean square displacement (MSD) for impermeable boundary, comparing computational and analytic results. At long times, particle displacements are uniformly distributed, with MSD $\Delta \vec {r}^2|_{\infty } = \sum l_i^2/6$ where $l_i = l_x,l_y,l_z$ as appropriate. Hence for $D_0= {1}\mathrm{{\upmu }m^{2}ms^{-1}}, l_x = 8\, \mu$m, $l_y=2l_x, l_z=13.75 l_x$, then $\Delta \vec {r}^2|_{\infty } = 2070\, \mu$m$^2$ as shown. (**c**) Comparison of intra, extra and overall MSD for impermeable boundaries as *T* increases.

Results are presented for a periodically repeating domain comprising a *3× 3* elongated permeable cell array, scaled to typical cardiomyocyte dimensions, as shown in Fig. 1a. In previous work^4,30^ we described a MCRW for a multi-compartment array with permeable barriers and jumps in diffusivity, compared with a 1D analytic and finite element solution. Here we modify the MCRW code to accommodate strain as outlined in methods. The domain of the fundamental element in Fig. 1 is extended to fill a much larger voxel as described below, since the code employed is designed for large complex tissue blocks. Note that for solutions using other methods or a simpler MCRW implementation, the total domain size is only required to be extended sufficiently to accommodate the dispersion of all particles during the strain cycle. The test geometry is intentionally grossly simplified from that of^30^ to allow comparison with analytic solutions and easy replication for other studies, e.g. to validate strain in a LBM or IB solver. All simulations assume identical intrinsic diffusivity $D_0 = {1}\mathrm{{\upmu }m^{2}ms^{-1}}$ of the cell and surrounding extracellular space to avoid confounding the results through changes in intrinsic diffusivity between intra and extracellular regions. At the extreme of infinite permeability and homogeneous intrinsic diffusivity, all barriers disappear, rendering the domain effectively unbounded. Conversely, for impermeable cells, diffusion occurs completely separately in cellular and extracellular compartments. These extremes bound the diffusion behaviour with finite permeability and provide checks on computational accuracy.

The model geometry block comprising cells and extracellular material is aligned with the imposed uniaxial strain, ensuring consistent alignement of diffusion tensor eigenvectors ($\vec {E1},\vec {E2},\vec {E3}$) with the principal axes (*z*,*y*,*x*). Thus the principal diffusion direction $\vec {E1}$ always points along one of the principal axes with $\vec {E2}$ and $\vec {E3}$ perpendicular in the remaining principal plane. This consistent orientation facilitates focussing on eigenvalue dynamics when analyzing strain effects. Assuming an isotropic medium (apart from membrane barriers), and a volume preserving strain, the deformation gradient tensor^42,43^ is diagonal:

5 $$\begin{aligned} \textbf{F}(t) = \begin{pmatrix} 1/\sqrt{h(t)} & 0 & 0 \\ 0 & 1/\sqrt{h(t)} & 0\\ 0 & 0 & h(t) \end{pmatrix} \end{aligned}$$

where *h*(*t*) defines the time varying strain. To investigate the effects of deformation on diffusion, we consider two distinct initial geometry configurations: the reference geometry configuration (C1) and the same geometry after a uniaxial (in *z*) volume preserving compression with $\varepsilon _z$ = -18%, (C2) as defined in Table 1.

### Characterising the nature of diffusion for the strain cycles of interest

Temporarily ignoring strain and permeability, diffusion in cells with membrane barriers is characterised by the relation between Einstein diffusion length scale $\langle x^2\rangle = 2Dt$, cell length scales and total diffusion time *T*. At extremely short *T*, few particles encounter barriers so diffusion appears homogeneous. At very long time scales, cell dimensions bound the total mean square displacement, (MSD = $\langle \Delta x^2+\Delta y^2 + \Delta z^2\rangle )$, so diffusion determined using Eq. (4) tends to zero. The interesting regime (especially for cDTI) is at intermediate times with sufficiently low permeability to restrict particle exchange.

To explore the effects of barriers, an analytic solution was obtained (see Supplementary Information, SI) for the MSD of random walkers released uniformly inside an impermeable cell undergoing deformation. This solution extends that for diffusion in a 1D, non-deforming slab [0, *L*] in Crank^1^, equation 4.56, via extension to 3D by tensor product of the static geometry eigenfunction expansion and replacement of constant diffusivity in the decaying exponential by an integral over time:

6 $$\begin{aligned} \left\langle {\left( {X_{i} - X_{{i,0}} } \right)^{2} } \right\rangle = & L_{i}^{2} \left[ {\frac{1}{6} - \frac{{16}}{{\pi ^{4} }}\sum\limits_{{n = 1}}^{\infty } {\frac{1}{{(2n - 1)^{4} }}} \exp \left( { - \frac{{(2n - 1)^{2} \pi ^{2} I_{{D,i}} (t)}}{{L_{i}^{2} }}} \right)} \right], \\ I_{{D,i}} (t) = & D_{0} \int_{0}^{t} h (s)ds,\;{\text{for }}i{\text{ corresponding to }}X,{\text{ or }}Y;I_{{D,i}} (t) = D_{0} \int\limits_{0}^{t} {\frac{{ds}}{{h^{2} (s)}}} ,\;{\text{for }}i = Z. \\ \end{aligned}$$

Here $X_{0,i}$ represents the start position of a walker and $X_{i}$ its position at a later time *t*. Capital letters denote *material coordinates*; the distinction between the *apparent* diffusion observed in the laboratory (i.e. in *laboratory* coordinates), versus the transformed diffusivity in material coordinates deforming with the body is explained in Methods and SI. The integral $I_{D,i}(t)$ depends on the strain in direction *i*, (derived in SI). In the unstrained case, $I_{D,i}(t)$ reduces to ${D_0}\,t$. For impermeable membranes, the 75% of particles initially inside our model geometry are trapped there, so Eq. (6) then captures the dominant, cellular contribution to the apparent diffusion (and provides an accuracy check for computational models).

For the model cell dimensions and $D_0$, the Einstein diffusion timescale in *x*, ($\tau _x = (l_x)^2/2D_0$) is only 32 ms given $l_x =$ 8*μ*m. Similarly $\tau _y =$ 128 ms whereas $\tau _z = 6050$ ms. Figure 1b plots the analytic *vs* computational evolution of the total MSD in the unstrained impermeable cell. Figure 1c shows the computationally derived evolution of the MSD for the intra- and extra-cellular spaces in the unstrained geometry with impermeable boundaries. In cDTI, relatively long diffusion times may be used, typically 1000 ms. At such comparatively long timescales, intracellular diffusion is evidently saturated in the *x-* and *y-*axes from the Einstein relation and the results in Fig. 1b,c. Extracellular diffusion is not saturated, but is characterised by a lower, orientation-dependent diffusivity.

**Table 1:** Cell and geometric parameters for configuration 1 (C1) and configuration 2 (C2).

Quantity	Symbol	Value/Expression
*Domain and base cell parameters*		
Number of cells per dimension	$N_x, N_y, N_z$	3, 3, 3 (array of $3\times 3\times 3$ cells)
*Reference geometry*	*C1, (configuration 1)*	
Cell size – (*x*-axis, *y*-axis, *z*-axis)	($l_x$, $l_y$, $l_z$)	($8\,\mathrm{{\upmu }m}$, $16\,\mathrm{{\upmu }m}$, $110\,\mathrm{{\upmu }m}$)
Block size – (*x*-axis, *y*-axis, *z*-axis)	($\Delta _x$, $\Delta _y$, $\Delta _z$)	($28.66\,\mathrm{{\upmu }m}$, $52.66\,\mathrm{{\upmu }m}$, $112\,\mathrm{{\upmu }m}$)
Cell–block boundary spacing	$\delta _{\text {block}}$	$1\,\mathrm{{\upmu }m}$
Intercellular spacing (target ECV)	*δ x*	$1.33\,\mathrm{{\upmu }m}$, calculated based on ECV
Extracellular volume fraction	ECV	*25%*
Block size formula	$\Delta _i$	$\Delta _i = N_i l_i + (N_i - 1)\delta _i + 2\delta _{\text {block}}$
ECV formula	–	$\displaystyle \text {ECV} = 1 - \frac{N_x N_y N_z\, l_x l_y l_z}{\Delta _x \Delta _y \Delta _z}$
*Compressed reference geometry*	*C2, (configuration 2)*	
C2 corresponds to geometry C1 compressed by the applied strains:		
Long axis strain	$\varepsilon _z$	*-18%*
Transverse strain (volume-preserving)	$\varepsilon _x = \varepsilon _y$	*10.43%*

### Simulation parameters

To accommodate applications beyond biological tissues, such as porous media or thermal conductivity problems, the permeability values explored cover the spectrum from impermeable (*κ = 0*) to fully permeable, ($\kappa \rightarrow \infty$). For specific application to cardiomyocytes, we focus on the physiologically relevant range, $\kappa = {0.001}{\mathrm{\upmu }}\textrm{m}\, \textrm{ms}^{-1}$ to ${0.05}{\mathrm{\upmu }}\textrm{m}\, \textrm{ms}^{-1}$, consistent with experimental observations in cellular tissues.

The cellular block structure shown in Fig. 1 is replicated as a periodically repeating geometry to fill a cubic voxel 2.8*×*2.8*×*2.8 mm$^3$, corresponding to a typical cDTI image voxel^23^. Particles move freely in global coordinates, while their interactions with the cellular geometry are determined by mapping to local coordinates within each block using modulo operations. The MCRW algorithm breaks each step into substeps when encountering boundaries of the block or the cell, maintaining trajectory continuity. This allows precise tracking of particle displacements in global coordinates.

**Fig. 2:**
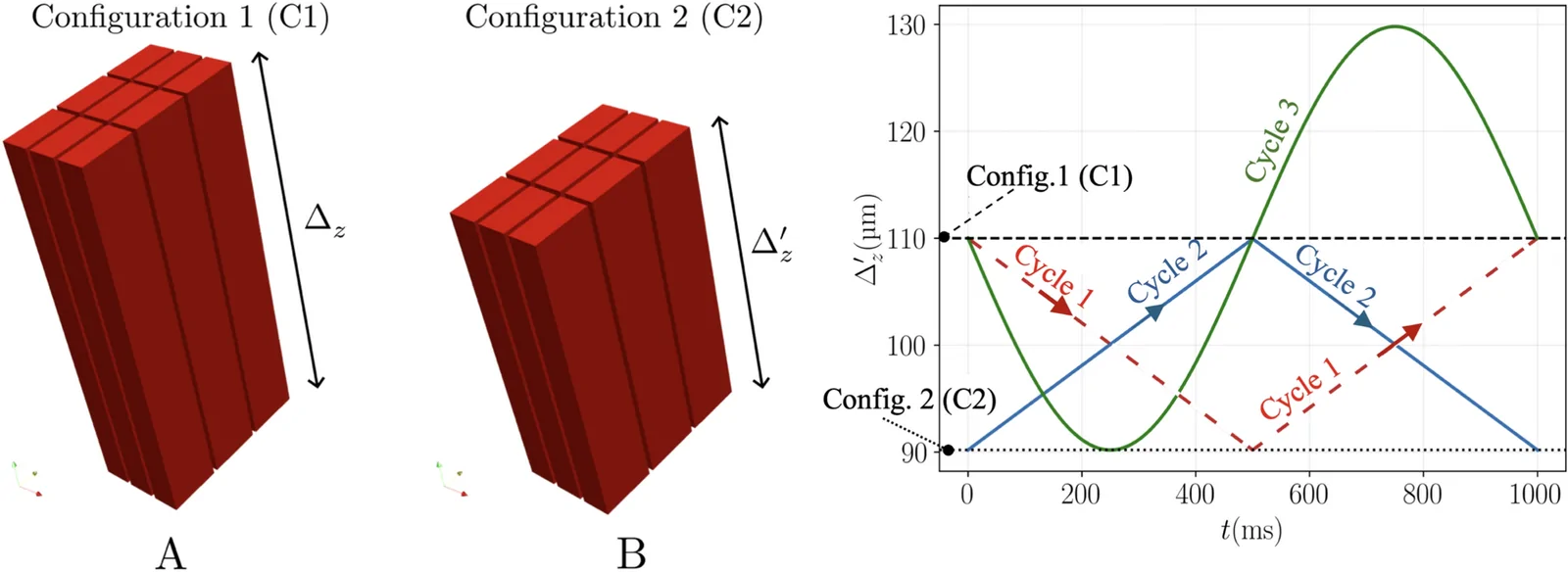
Left: A) 3D illustration of configuration 1 (C1), which undergoes linear compression (cycle 1) and sinusoidal strain (cycle 3) along the *z*-axis ($\varepsilon _z = -18\%$), (with expansion in the *x*-*y* plane to preserve volume). (B) 3D illustration of configuration 2 (C2), obtained by compressing the initial geometry C1 along the *z*-axis to its maximum strain ($\varepsilon _z = -18\%$). Right: Temporal profiles of the strain cycles: 1 - linear compression (dashed-red); 2 - linear expansion (solid-blue); 3 - sinusoidal strain (solid-green). Geometry C2 undergoes a greater expansion strain along the *z*-axis ($\varepsilon _z = +21.95\%$) to recover the original dimensions of C1 at $t = {500}ms$.

For strain modeling, three different cyclic patterns of uniaxial strain are implemented. Each lasts 1000 ms and represents compression, expansion, and sinusoidal strain in the principal long axis (*z*-axis) of the domain, as summarised in Table 2. Cycles 1 and 3 start with initial condition (IC) in the unstrained or reference configuration, denoted C1. Cycle 2 starts with initial condition at the point of maximum compressive strain, $\varepsilon _z = -18\%$, denoted configuration C2 and undergoes a maximum expansion of $\varepsilon _z = 21\%$ to recover the geometry of C1 at the cycle half way time, *t =T/2 =* 500 ms. These strain values are in line with those measured in cardiomyocytes along their principal axes^38^. The strain cycles and interplay between geometries wih intial condition C1 and C2 are illustrated in Fig. 2.

**Table 2:** Summary of simulation setups and strain patterns for geometries C1 and C2.

Cycle	Initial geometry	Strain setup
1: Compression	C1	Linear compression-expansion cycle: C1*→* C2 *→* C1. $$\epsilon _z(t) = {\left\{ \begin{array}{ll} -0.18 \dfrac{t}{500}, & 0 \le t \le 500\,\text {ms},\\ -0.18 \dfrac{1000 - t}{500}, & 500 < t \le 1000\,\text {ms}, \end{array}\right. }$$
2: Expansion	C2	Linear expansion-compression cycle: C2*→* C1 *→* C2. $$\epsilon _z(t) = {\left\{ \begin{array}{ll} 0.2195 \dfrac{t}{500}, & 0 \le t \le 500\,\text {ms},\\ 0.2195 \dfrac{1000 - t}{500}, & 500 < t \le 1000\,\text {ms}, \end{array}\right. }$$ (At $t = 500\,\text {ms}$, C2 matches original C1 geometry)
3: Sinusoidal	C1	Sinusoidal long-axis strain cycle $\epsilon _z(t) = -0.18 \sin \!\left( \dfrac{2\pi t}{1000}\right)$ (At $t = 250\,\text {ms}$: C1 matches initial C2 geometry)

The geometry and simplified strain cycles are chosen so that simulation results can be related to practical cDTI measurements, in which the diffusion signal reflects the ensemble average behavior of water molecules within each imaging voxel. For all simulations, a homogeneous distribution of 5*×* 10$^5$ random walkers is initialized in an isotropic cubic voxel (2.8$^3$ mm$^3$, as described), sampling both intracellular and extracellular spaces to yield a diffusion tensor representative of the microstructural characteristics. Walkers are stepped using fixed time steps of 0.1 ms with a constant homogeneous diffusivity throughout ($D_0= {1}\mathrm{{\upmu }m^{2}ms^{-1}}$). Further details of the MCRW method including treatment of permeability are given in^4–6^, whilst validation tests are detailed in Methods and in SI. Results of particular note in SI show that the random walk and geometry deformation steps can be decoupled. There is no loss of accuracy in reducing geometry update frequency to 1% of that for the random walk, i.e. the strain regimes of interest appear quasi-steady. Therefore the detailed analysis and validation of the MCRW treatment of permeability in^4^ is expected to carry over, though a direct computational validation is also included in SI.

### Diffusion in the unstrained initial geometries

**Fig. 3:**
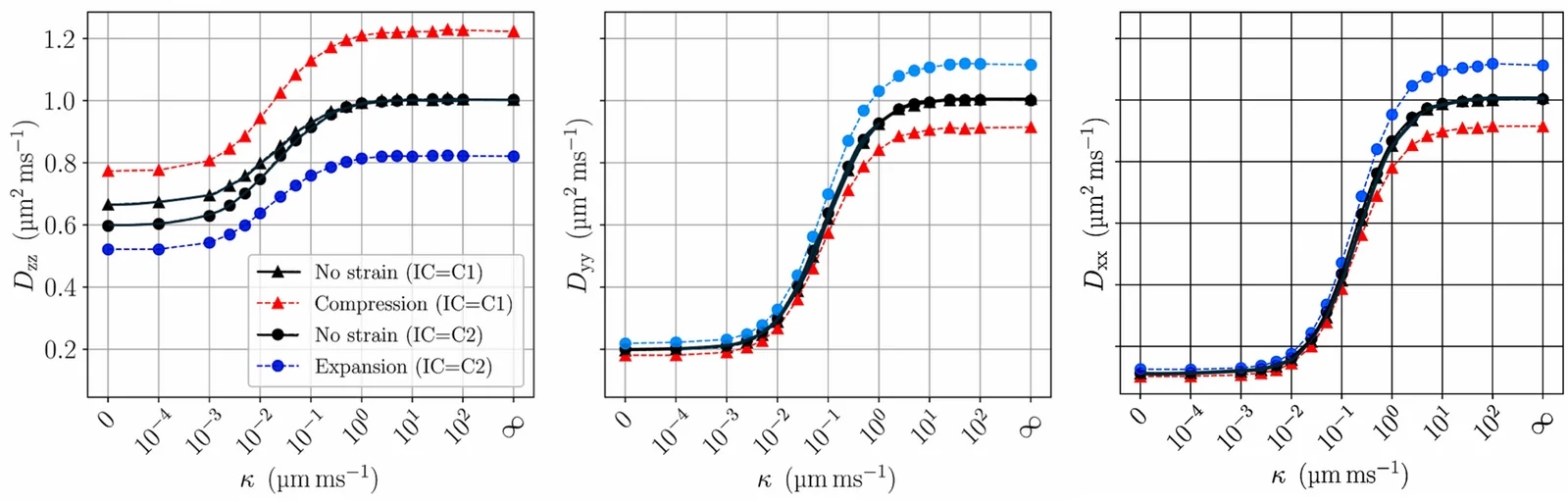
Effect of strain on apparent diffusivity along principal axes ($D_\text {zz}$, $D_\text {yy}$, $D_\text {xx}$) as a function of membrane permeability (*κ*). Triangle markers reference simulations using geometry C1 as initial condition, with dashed connectors for the compression cycle 1, solid connectors, no strain. Circle markers represent geometry C2 as initial condition with dashed connectors for expansion cycle 2, solid connectors no strain. A clear transition is observed from no or weakly restricting barriers, corresponding to $\kappa \ge {1}{\mathrm{\upmu }}\textrm{m}\, \textrm{ms}^{-1}$, to barrier restricted ($\kappa < {1} {\mathrm{\upmu }}\textrm{m}\, \textrm{ms}^{-1}$) diffusion, with strain effects becoming attenuated as permeability decreases.

In the absence of strain and with low or zero permeability, both initial geometry configurations C1 and C2 demonstrate distinct apparent diffusivity coefficients along different axes. The increased dominant diffusion along the z-axis $D_{\text {zz}}$ for configuration C1 reflects its greater initial elongation compared with C2 (see Fig. 3), whilst the low diffusivity values in *x*, *y* with $D_{\text {yy}} > D_{\text {xx}}$ for both C1 and C2 reflect the fully saturated diffusion at *T=1000* ms in the cellular compartment (which holds 75% of particles) and the increased obstruction of barriers in *x* compared to *y* directions. When the different cyclical strain patterns are introduced, these baseline apparent diffusivity coefficients are modified differently depending on strain function. For infinite permeability ($\kappa =\infty$) and no strain, Fig. 3 shows diffusion is homogeneous with both geometries (C1 - triangle markers; C2 - circle markers) converging to the initial isotropic diffusivity coefficient $D_{\text {zz}}=D_{\text {yy}}=D_{\text {xx}}= D_0 ={1}\mathrm{{\upmu }m^{2}ms^{-1}}$ as $\kappa \rightarrow \infty$.

### Effects of strain

(i) $\boldsymbol{\kappa } \rightarrow \boldsymbol{\infty }$ As outlined in Methods (Eq. (31) and see SI) for the limiting case of $\kappa = \infty$, the analytically derived diffusion coefficients for cycle 1 (compression: C1*→*C2*→*C1) are respectively: $D_{zz}(T) = 1.22{D_0},\,\,D_{xx} = D_{yy} = 0.91{D_0}$, and for cycle 2 (expansion: C2*→*C1*→*C2) are $D_{zz}(T) = 0.825{D_0},\,\,D_{xx} = D_{yy} = 1.106{D_0}$, in agreement with the computational results for $\kappa = \infty$ in the figure.
(ii) *κ = 0* **(impermeable cells)**. Considering only the cellular compartment, when membrane barriers are introduced, Eq. (6 predicts the MSD of walkers in both *x* and *y* directions to be fully saturated, with and without strain, at 10.67 and 42.67 *μ*m$^2$ respectively, corresponding to $D_{xx}$ of 0.005 and $D_{yy}$ of 0.021 times $D_0$. This agrees perfectly - to this precision - with computations using 5 $\times 10^5$ intracellular particles and *dt=0.1* ms. With strain cycle 1, Equation 6 gives an increase in $D_{zz}/D_0$ from 0.569 to 0.641 (computation gives 0.568 to 0.640). Note that for this cycle, the relative increase in apparent diffusivity in *z* due to strain, namely *0.641/0.569 = 1.13* is far less than for infinite permeability $D_{zz}/D_0 = 1.22$.

For the extracellular compartment containing 25% of the walkers, computations (see SI) predict effective diffusivity ratios $D_{ii}/D_0$ of 0.43, 0.72 and 0.95 respectively for *i* corresponding in turn to *x*, *y* and *z* orientations. Since intracellular diffusion in *x* and *y* directions is saturated, the majority of the diffusivity $D_{xx},\,D_{yy}$ is due to particles in the extracellular space. Thus for example $D_{xx}/D_0 \approx 0.25 \times 0.43 = 0.11$ as shown.

*Full range of permeability* Referring to Fig. 3, strain alters the apparent diffusivity across the entire permeability range. For cycle 1, where the medium is first compressed (*ε <0*), the apparent principal diffusion coefficient $D_{zz}$ is increased; for cycle 2 expanding the medium (*ε >0*) before recompressing produces the opposite effect. Quantitatively, for $\kappa =\infty$, geometry C1 under *z*-axis compression exhibits a 22% increase in $D_{zz}$, while geometry C2 under expansion shows a -18% decrease in $D_{zz}$. For the transverse *x* and *y* axes, we observe the converse for the secondary coefficients $D_{xx},D_{yy}$, at reduced effect due to volume preservation constraints: compressing geometry C1 changes $D_{xx},\, D_{yy}$ by -9%, while expanding geometry C2 increases these by +11%. In all configurations, compression has a greater effect than expansion on diffusion in the strain direction.

Overall, Fig. 3 shows that for permeability values $\kappa >{1}{\mathrm{\upmu }}\textrm{m}\, \textrm{ms}^{-1}$, diffusion remains effectively homogeneous, almost identical to the fully permeable case. Below this threshold ($\kappa <{1}{\mathrm{\upmu }}\textrm{m}\, \textrm{ms}^{-1}$), diffusion appears orientation dependent. As permeability decreases from $\kappa = {1}{\mathrm{\upmu }}\textrm{m}\, \textrm{ms}^{-1}$ to $\kappa = {0.001}{\mathrm{\upmu }}\textrm{m}\, \textrm{ms}^{-1}$ diffusing particles experience greater restriction, particularly in *x* and *y* directions. Since most particles reside initially in the cells, much of the behaviour follows the MSD plots of Fig. 1, and Equation 6. At very low permeabilities and the extended time of 1000 ms, intracellular diffusion in *x* and *y* becomes saturated. The extracellular compartment then becomes the dominant contribution to MSD in the transverse plane, explaining why $D_{xx}$ and $D_{yy}$ converge to *≈* 0.1 ${D_0}$ and $\approx 0.2 {D_0}$ respectively, and strain effects in *x* and *y* appear negligible in absolute terms.

**Fig. 4:**
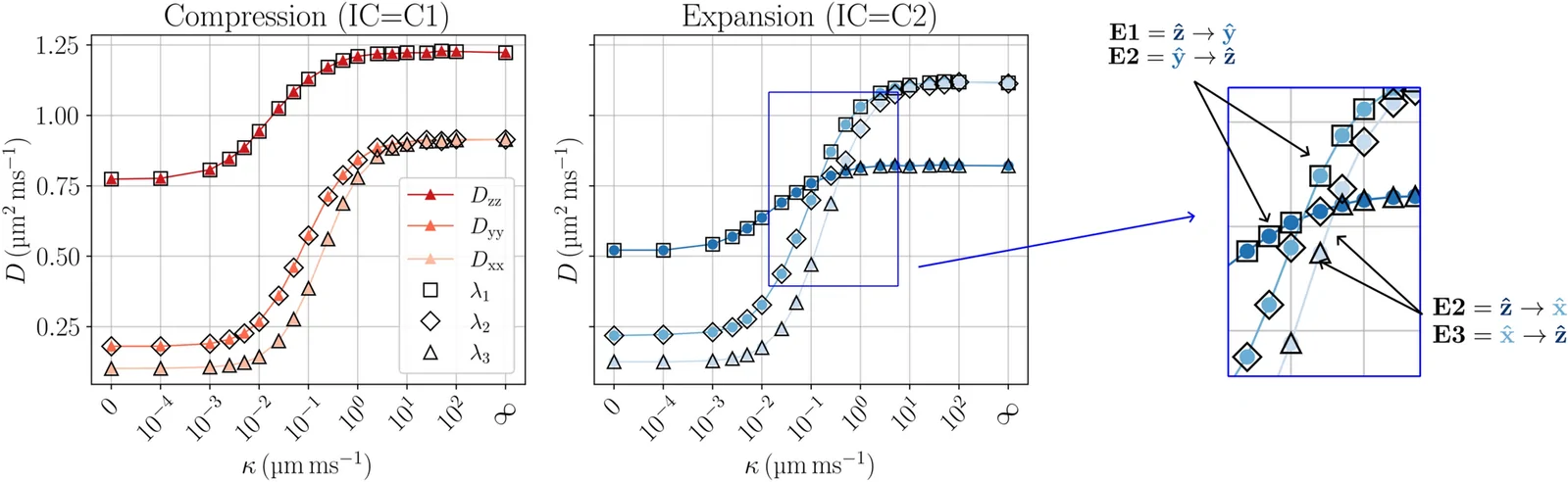
Eigenvalues and apparent diffusion tensor parameters for geometry C1 in the compression cycle 1, and geometry C2 in expansion cycle 2 across a full range of permeability values. Varying intensity of solid markers is used to distinguish apparent diffusivity parameters ($D_\text {zz}$, $D_\text {yy}$, $D_\text {xx}$), while the eigenvalues ($\lambda _1$,$\lambda _2$,$\lambda _3$) are illustrated as unfilled markers. Geometry C2 exhibits an eigenvector reordering phenomenon in the transition region between barrier-restricted and free diffusion regimes. Around $\kappa ={0.1}{\mathrm{\upmu }}\textrm{m}\, \textrm{ms}^{-1}$, the principal diffusion direction shifts from the *z*-axis to the *y*-axis, followed by a second reordering at $\kappa = {1}{\mathrm{\upmu }}\textrm{m}\, \textrm{ms}^{-1}$, where diffusion along the *x*-axis exceeds that along the *z*-axis. This intermediate region represents a state where strain and permeability effects effectively counterbalance each other’s influence on diffusion anisotropy. In contrast, geometry C1 shows no reordering, and the eigenvectors remain aligned across all permeability values.

Interestingly, as shown in Fig. 4, during the transition from low permeability ($\kappa = {0.001}{\mathrm{\upmu }}\textrm{m}\, \textrm{ms}^{-1}$ to high permeability, ${1}{\mathrm{\upmu }}\textrm{m}\, \textrm{ms}^{-1}$), a critical equilibrium point appears for the expansion cycle 2. In this permeability regime, the reduction in diffusivity due to expansion in the long (*z-*) axis compensates for the increase caused by compression in the short (*x* and *y* ) axes. Specifically, we note two distinct eigenvector reorderings as permeability increases: first, at approximately $\kappa ={0.1}{\mathrm{\upmu }}\textrm{m}\, \textrm{ms}^{-1}$, the principal diffusion direction shifts from the long axis *z* to the *y* axis; subsequently, at $\kappa ={1}{\mathrm{\upmu }}\textrm{m}\, \textrm{ms}^{-1}$, the effective diffusion in the *x*-axis surpasses that in the *z*-axis, causing the second eigenvector to reorder from *z* to *x*.

**Fig. 5:**
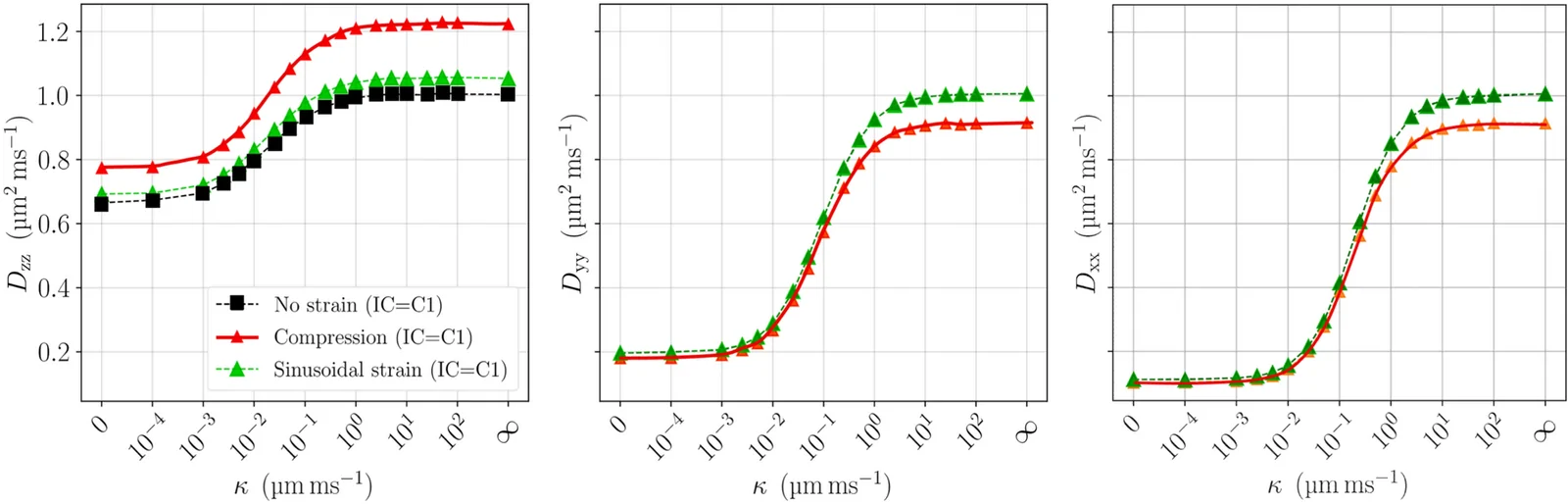
Comparison between linear compression and sinusoidal strain patterns using geometry C1, illustrating apparent diffusivity coefficients $D_{\text {zz}}$, $D_{\text {yy}}$, and $D_{\text {xx}}$ versus barrier permeability *κ*. Despite the balanced compression-expansion cycle in sinusoidal strain (see Fig. 2), a net positive diffusivity increase of approximately *+5%* persists along the *z*-axis, with minimal changes along the *x* and *y* axes, demonstrating the asymmetric influence of compression versus expansion on diffusion properties.

Extending to a balanced strain cycle, Fig. 5 compares a linear compression then expansion versus a sinusoidal strain cycle using as initial geometry configuration 1 (C1). In the sinusoidal cycle, compression occurs in the first quarter to reach geometry C2. This is followed by expansion over a half-cycle and terminates with a quarter cycle compression back to C1, with the cycle attaining identical maximum positive and negative strains ($\epsilon =\pm 18\%$). Despite being a balanced compression-expansion cycle, the apparent diffusivity is affected, though more weakly. Strain effects are most evident in the long axis (*z*) with an increase of $D_{\text {zz}}$ of approximately *+5%* for all permeability values. Similarly to Fig. 3, due to volume preservation, the short axes (*x* and *y*) experience a corresponding sinusoidal strain but with reduced magnitude, resulting in minimal diffusivity changes (with approximately *+0.05%* increase) along both dimensions.

**Fig. 6:**
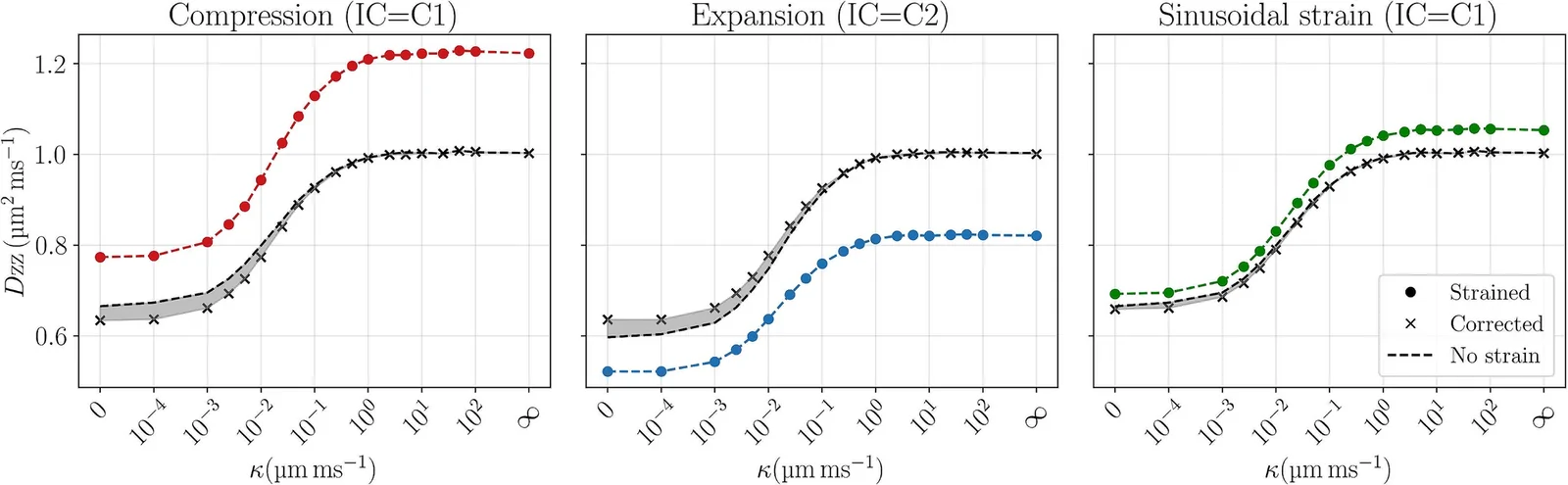
Comparison between the corrected strained apparent diffusivity coefficients $D_{\text {zz}}$ and the $D_{\text {zz}}$ without strain considering the previous strain patterns and the range of permeability values. The strain values are corrected assuming a Gaussian diffusion model from Equation 25, consequently, correction accuracy improves as permeability increases toward the Gaussian diffusion regime.

Applying standard strain correction methods derived for homogeneous diffusion by Reese et al^35,39^ (see equation Equation 8 in Methods), shows systematic errors in recovering the true diffusion properties $\textbf{D} = D_0\textbf{I}$ in the low permeability (barrier-dominated) regime. Fig. 6 illustrates corrected strain values along the long (*z*) axis, where strain effects are most pronounced. With low permeability, the correction factor is consistently too large, for both geometry configurations and all strain types examined. The magnitude of overcorrection directly correlates with the area under the strain function curve, being greater in unbalanced strain patterns dominated by compression or expansion; balanced (sinuosidal) strain shows reduced overcorrection. Quantitatively, for the impermeable case (*κ =0*), the compression-strained $D_{zz}$ is overcorrected by *4.7%*, while the expansion-strained $D_{zz}$ is overcorrected by *6.5%*. Even sinusoidal strain where strain effects are reduced shows a measurable *0.9%* overcorrection relative to no strain. The over correction reflects that at low permeability and longer diffusion times, the MSD of particles is limited. Since the strain correction is based on unbounded diffusion, it therefore progressivley over-corrects. For example, applying the unbounded diffusion correction factor of cycle 1 (*≈ 1.22*) to the intracellular particles would predict the unstrained *z* diffusivity should increase from the theoretical 0.569 $D_0$ to 0.694 whereas Equation 6 gives the true increase of only 12.7% to 0.641. Factoring in the relative contribution of the extracellular portion explains the nearly 5% overall over-correction. Although these errors limit the correction accuracy in complex media with semi-permeable barriers, for the moderate strains considered, it is reassuring that the scale of correction appears relatively minor.

## Discussion

In an unbounded medium, effects on the apparent diffusion tensor are independent of the duration of the strain cycle. At the other extreme, where parts of the domain are enclosed by impermeable boundaries, diffusivity values computed from the MSD are sensitive to cycle duration. Analysis of diffusion in an impermeable cuboidal cell during a time varying deformation yielded Equation 6, derived in SI. With this, the effects of any time-varying strain cycle on the apparent diffusion can be predicted. When applied to the cycles considered, excellent agreement was found with numerical simulations.

Computational methods are needed to analyze strain effects in non-homogenous regions with semi-permeable barriers in 3D. The simulations presented here were motivated by the need to investigate possible confounding effects of strain in cDTI, specifically imaging using the stimulated echo acquisition mode (STEAM) sequence^37^. STEAM imaging encodes diffusion over a complete cardiac cycle (*∼*1000ms) with a typical spatial resolution of  2.8mm in plane, motivating our experimental parameters. Acquisitions are typically triggered to end systole (expansion) or end diastole (compression), but may be acquired at mid-contraction (sinusoidal strain), hence our choice of configurations. However in order to facilitate theoretical analysis and for easy replication in other studies, the model cardiomyocyte geometry is deliberately idealised. The combination of model cell dimensions and cycle time considered mean that our simulations occupy the intermediate temporal regime of diffusion in the presence of barriers. As evident from both theoretical formulae and the MSD plots of Fig. 1, at extremely short times, few particles encounter barriers and diffusion would appear isotropic irrespective of permeability. At long times, with impermeable barriers the response of the cellular compartment tends towards saturation, increasing the relative contribution of the extracellular compartment.

With membrane permeability, two distinct diffusion regimes have been identified for the model geometry: for $\kappa > {1}{\mathrm{\upmu }}\textrm{m}\, \textrm{ms}^{-1}$, diffusion follows that in an unbounded domain. As *κ* diminishes, diffusion becomes ever more highly restricted at longer times, with $D_{\text {zz}}> D_{\text {yy}} > D_{\text {xx}}$ corresponding to the decreasing cell length scales $l_z> l_y > l_x$. From a physical perspective, the difference between these permeability regimes can be understood by examining the relationship between *κ* and particle exchange. Assuming uniform concentration within compartments, the mean exchange time $\tau _{\text {exchange}}$ is related to *κ* by $\tau _{\text {exchange}} \approx (V)/(S\kappa )$ where *V* is the cell volume and *S* is the surface area^44^. For the simplified domain here, the cell surface to volume ratio is $V/S={2.5}\mathrm{{\upmu }m}$, which means $\tau _{\text {exchange}} \approx 2.5/\kappa$. This relationship explains the transition between diffusion regimes: for $\kappa ={1}{\mathrm{\upmu }}\textrm{m}\, \textrm{ms}^{-1}$, $\tau _{\text {exchange}}={2.5}ms$, allowing particles ample time to exchange between compartments during our simulation time $T={1000}ms$, approximating behavior in an unbounded homogeneous medium. Conversely, for $\kappa ={0.001}{\mathrm{\upmu }}\textrm{m}\, \textrm{ms}^{-1}$, $\tau _{\text {exchange}}={2500}ms$, exceeding *T* and leading to barrier restricted diffusion as particles remain predominantly confined to their original compartments. In biological tissues and in particular in muscle cells, permeability often ranges between ${0.01}{\mathrm{\upmu }}\textrm{m}\, \textrm{ms}^{-1}$ and ${0.05}{\mathrm{\upmu }}\textrm{m}\, \textrm{ms}^{-1}$, indicating that in this application diffusion is highly restricted^45–47^. The findings on how permeability affects diffusion, reported here for the simplified model domain, align with previous simulations in more realistic unstrained substrates^30^.

At high values of permeability ($\kappa >{1}{\mathrm{\upmu }}\textrm{m}\, \textrm{ms}^{-1}$), membranes appear unrestrictive and strain effects follow the pattern predicted by the equation:

7 $$\begin{aligned} \textbf{D}_{\textrm{lab}}(t) = \textbf{F}(t)\textbf{D}_{\textrm{material}}(t)\textbf{F}(t)^{{\!\top }}, \end{aligned}$$

where superscript $\mathrm{T}$ denotes transpose. This relation and the meaning of subscripts *lab* and *material* are described in the methods section: briefly *lab* refers to what is actually measured in the (spatially fixed) coordinate frame of the laboratory, versus what would be measured in a (*material*) frame deforming with the material. It is also shown that the distinction vanishes for a complete cycle so that:

8 $${\mathbf{D}}_{{{\mathrm{lab}}}} (T) = \frac{{D_{0} }}{T}\int\limits_{0}^{T} {{\mathbf{F}}^{{ - 1}} } (s){\mathbf{F}}^{{ - {\!\top }}} (s){\mkern 1mu} ds = {\mathbf{D}}_{{{\mathrm{material}}}} (T),$$

(Equation 7 represents the more general form of the equation presented in^35,39^). In the low permeability regime, the above equations fail to fully predict the effects of strain (Fig. 6) given that in an impermeable cell, strain effects progressively weaken; the intracellular diffusion is quantifiable using Equation 6. However for the biologically relevant moderate strains (of *O(20%)*) considered, the error is relatively low. The trend is well predicted - notably compression increases effective diffusivity while expansion reduces it. Results reveal an asymmetric response where compression produces stronger diffusivity changes than expansion of equivalent magnitude. This reflects the *1/(1+ε )* term, where *ε* is the strain in *z* that appears in evaluating the $D_{zz}$ component in Equation 8 for strain linearly varying in time, with *ε = -0.18* for the compression cycle and positive for the expansion cycle. Thus the strain-induced changes are larger in magnitude for negative strain values (compression, *ε <0*) than under expansion (*ε >0*). This asymmetry also appears in the sinusoidal cyclic strain simulations (Fig. 2), where equal periods of compression and expansion always yield a positive non-zero net effect on diffusivity. Similar findings have been observed in cardiac diffusion tensor imaging when comparing diastolic (mostly compression) versus systolic (mostly expansion) measurements^48,49^.

For the expansion cycle (cycle 2), we observed a significant eigenvalue reordering. This eigenvalue reordering occurs at a specific permeability *κ* where the expansion-induced reduction in the long axis (z-direction) counterbalances the compression-induced increases in the short axes (*x* and *y* directions). The expansion-induced diffusivity reduction along the principal (*z*) diffusion axis precisely offsets the compression-induced enhancement along the short axes (*x*-*y* plane). This creates a configuration where strain and permeability effects counterbalance, minimizing diffusion anisotropy. The phenomenon appears only in cycle 2 as the cyclic compression in cycle 1 enhances diffusivity along the *z*-axis, further amplifying anisotropy. With our geometry C2, where $l_y > l_x$, complete isotropy ($\lambda _1=\lambda _2=\lambda _3$) remains unachievable due to the inherent geometrical anisotropy. Theoretically, if $l_y = l_x$, the strain-permeability mechanism might be used to achieve diffusion isotropy despite the underlying structural anisotropy ($l_z>l_x=l_y$). However at typical expected cardiomyocyte membrane permeabilities (on the order of $\kappa = 0.01 \mu$m ms$^{-1}$), results suggest that there may be some loss of anisotropy due to strain, but eigenvector swapping is unlikely, and that the standard strain correction methods do somewhat over-estimate the magnitude of the effect of strain.

The correction methodology relies on assumptions of homogeneous diffusion in an unbounded domain. It provides an accurate correction when permeability values are sufficiently high ($\kappa \ge {1}{\mathrm{\upmu }}\textrm{m}\, \textrm{ms}^{-1}$), placing the system near the free diffusion regime. With lower barrier permeability, the correction accuracy diminishes: reduced *κ* attenuates strain effects, causing the correction factor to overcorrect for strain effects. These findings correspond with experimental observations in cardiac diffusion tensor imaging^10^, where this same strain-correction model has been shown to amplify the contribution of strain to measured diffusion parameters.

While we based our experiments on in vivo cardiac diffusion tensor imaging, where strain during the measurement is of particular relevance, our MCRW model can be deployed to understand the effects of strain and membrane permeability regardless of application. Of potential benefit for future studies is our finding from convergence analysis (SI) that for the strain values considered *<20%*, strain time steps $\Delta t_\epsilon$ can be much larger than diffusion time steps $\Delta t_D$, reducing computational costs by minimizing the need to store multiple deformed geometries.

### Conclusion

The analytic solution for a cuboidal, impermeable cell (derived in SI), allows strain effects on diffusion to be predicted, though in complex media with semi-permeable membranes computational methods are required. We have shown that the MCRW method successfully models diffusion in deforming media with complex geometries, capturing barrier effects missed by analytical models. Applied to a dynamically altering geometry embodying features of cardiac muscle tissue, analysis and MCRW computations enabled the effects of strain on diffusion in semi-permeable media to be quantified for the first time. This study extends previous analysis for an unbounded, homogeneous domain, showing that the effects of strain are significantly attenuated as permeability decreases. Cyclical strain patterns consistently affect the apparent diffusion in impermeable, semi-permeable, and fully-permeable media. Compression (negative strain values) increases the effective diffusivity in the principal strain direction while expansion (positive strain values) decreases it, with compression producing larger magnitude changes than equivalent expansions across all permeability values. This explains why sinusoidal strain patterns with equal compression/expansion periods yield a small but net effect of increased diffusivity.

Using the standard strain correction method for homogeneous Gaussian diffusion is accurate when used as it was intended but overcorrects for low permeability values where strain effects are reduced. These findings have potential impact in a number of areas and we have performed simulations with parameters consistent with in vivo cardiac diffusion tensor imaging using stimulated echo acquisition mode methods. Strain has a particular relevance in stimulated echo based measures of diffusion in the heart as strain potentially adds systematic biases to the measured diffusion tensor which is used to infer the microscopic structure of the heart. More generally this work elucidates the impact of strain on diffusion in restricted media, paving the way for better interpretation and understanding not only in cardiac diffusion tensor imaging but in other applications where deformation significantly impacts diffusion measurements. In terms of application to a specific problem, our model is limited to a highly idealised geometry. However the methodology and findings presented could provide a foundation for developing more models of greater complexity in geometry, structure, strain functions and barrier characteristics, accurately distinguishing between strain-induced changes and intrinsic tissue or material properties in complex biological or other systems.

## Methods

### MCRW algorithm

The MCRW approach to model diffusion is well known, while adaptations capable of dealing with permeable boundaries are described in^4–6^. In essence the MCRW models diffusion by determining the spread of a large set of particles undergoing a random walk. Specifically, each random walker steps in the domain according to:

9 $$\begin{aligned} {y}(t+\Delta t) = {y}(t) + {\eta } \end{aligned}$$

where *y*(*t*) and ${y}(t+\Delta t)$ are the particle positions before and after the time step *Δ t*, and *η * is a random step vector. Typically $\eta \sim \sqrt{2D\Delta t}\mathcal {N}(0,1)$; alternatively, by the Central Limit Theorem^50^, *η * can sampled from other distributions, such as random fixed steps $\pm \ell = \pm \sqrt{2D\Delta t}$. Using fixed steps, though of slower convergence simplifies the analysis as described below.

### Diffusion tensor reconstruction

Equation 4 defines the diffusion tensor in terms of the displacement covariance matrix. For computational modelling we can align the coordinate axes with the principal axes of $\textbf{D}$, in which case the off-diagonal terms vanish and the diagonal terms correspond with the eigenvalues. (This is unlike *in vivo* where axes do not generally align and all 6 independent terms need to be measured). In the MCRW method, the coordinates of each walker $\boldsymbol{y}$ are then updated by a random vector $\boldsymbol{\eta }$, scaled to match the direction-dependent diffusivity on each principal axis. Finally the diffusion coefficients on each principal axis *i* are calculated from the relevant MSD and elapsed time *T* as $D_{ii} = \langle (\Delta y_i)^2\rangle /(2T)$

### Characterising deformation

In analysing a deformation, it is convenient to distinguish between *material* (also called *reference*) coordinates $\textbf{X}$, and *laboratory* (also called *spatial*) coordinates^42,43^. Here we use $\boldsymbol{y}$ to denote lab coordinates, rather than lower case $\textbf{x}$, (the latter is a frequently used notation for lab coordinates but makes reading difficult). Material coordinates are attached to points in the material and move with the material as it deforms. By contrast lab coordinates are fixed in space. At time *t*, the deformation map $\chi (\textbf{X},t)$ relates the material coordinate $\textbf{X}$ of a point to its corresponding lab coordinate $\boldsymbol{y}$:

10 $$\begin{aligned} \boldsymbol{y} = \chi (\textbf{X},t) \end{aligned}$$

From the chain rule, infinitesimal changes in a material vector $d\textbf{X}$ are related to corresponding changes $d\boldsymbol{y}$ in lab coordinates by the deformation gradient $\textbf{F}(t) = \nabla _X\chi$:

11 $$\begin{aligned} d\boldsymbol{y}(t) = \textbf{F}(t)\,d\textbf{X} , \qquad \textbf{F}(t) = \frac{\partial \chi (\textbf{X},t)}{\partial \textbf{X}}. \end{aligned}$$

If at some time *s* we have $d\boldsymbol{y}(s) = \textbf{F}(s)\,d\textbf{X}$ and at some later time *t*, $d\boldsymbol{y}(t) = \textbf{F}(t)\,d\textbf{X}$, then eliminating $d\textbf{X}$ gives:

12 $$\begin{aligned} d\boldsymbol{y}(t) = \textbf{F}(t)\,(\textbf{F}(s)^{-1} d\boldsymbol{y}(s)) = {\Phi }(t,s) d\boldsymbol{y}(s). \end{aligned}$$

Here $\Phi (t,s) = \textbf{F}(t)\,\textbf{F}(s)^{-1}$ is the propagator which transports a displacement in the laboratory frame $d\boldsymbol{y}$ from time *s* to some later time *t*. We restrict attention to pure deformations (no translation) that are homogeneous in space, i.e. globally affine. In this case we can omit the differentials and write:

13 $$\begin{aligned} \boldsymbol{y}(t) = \textbf{F}(t)\,\textbf{F}(s)^{-1}\,\boldsymbol{y}(s) = {\Phi }(t,s) \boldsymbol{y}(s) \end{aligned}$$

### Strain effects on Gaussian diffusion: random-walk formulation

Continuum deformation theory^42,43^ is usually employed to determine the effects of deformation on diffusion. Here we show an alternative approach that aligns directly with the random walk model. by considering the strain applied incrementally to a sequence of small random displacements. Under a time-varying deformation, these random displacements are advected with the material, changing their cumulative displacement. The net effect is to alter the pattern of diffusive spreading. In cDTI measurements for example, we initially tag each point in the initial or reference configuration with a phase. The diffusion-induced loss of signal allows the diffusion tensor to be determined, from which properties of the tissue including structural anisotropy can be inferred. However the cyclic compression and expansion of the heart alters the observed diffusion and we wish to determine the magnitude of such effects. We assume the intrinsic diffusion tensor, $\textbf{D}_0$, of the material is not altered and seek to determine purely the effects of deformation.

Denote the diffusion tensor measured in the material coordinates at time *t* as $\textbf{D}_{\textrm{material}}(t)$, and that measured at the same time in the laboratory coordinate system as $\textbf{D}_{\textrm{lab}}(t)$. To highlight the effect of deformation we take the intrinsic diffusion as isotropic, $\textbf{D}_0 = D_0\textbf{I}$, but the same approach applies to an anisotropic diffusion tensor.

Since a discrete sequence of infinitesimal increments can approximate a smooth deformation history, the effect of deformation on the spatial mapping of a particle can be written as:

14 $$\begin{aligned} \boldsymbol{y}_n \;=\; \textbf{F}_c(n)\,\textbf{X}_n, \qquad \textbf{F}_c(n) \;\equiv \; \textbf{F}_{\textrm{inc},n}\,\textbf{F}_{\textrm{inc},n-1}\cdots \textbf{F}_{\textrm{inc},1}. \end{aligned}$$

To incorporate the effect of diffusion using the MCRW approach with fixed step length *ℓ* for each axis, the spatial position of each point $\boldsymbol{y}$ in a cloud of particles is updated from time step $j \rightarrow j{+}1$ through:

- A random isotropic displacement $\boldsymbol{\eta }_{j+1}$ with variance $\ell ^2$  applied to each axis;
- An incremental deformation $\textbf{F}_{\textrm{inc},j+1}.$

Therefore the position of each particle in a cloud of particles evolves in the laboratory frame as:

15 $$\begin{aligned} \boldsymbol{y}_{j+1} = \textbf{F}_{\textrm{inc},j+1}\boldsymbol{y}_j + \boldsymbol{\eta }_{j+1}, \quad \text { where }\quad \mathbb {E}[\boldsymbol{\eta }_{j+1}\boldsymbol{\eta }_{j+1}^{\!\top }] = \ell ^2\textbf{I},\qquad \end{aligned}$$

and superscript *⊤* represents the transpose. Equation 15 captures both the advection of the particles and the change in apparent diffusivity through the cumulative operation of the incremental deformation on the random steps. The spatial covariance $\boldsymbol{\Sigma }_j = \mathbb {E}[\boldsymbol{y}_j\boldsymbol{y}_j^{\!\top }]$ evolves at each step as:

16 $$\begin{aligned} \boldsymbol{\Sigma }_{j+1} = \textbf{F}_{\textrm{inc},j+1}\boldsymbol{\Sigma }_j \textbf{F}_{\textrm{inc},j+1}^{\!\top } + \ell ^2\textbf{I}. \end{aligned}$$

Corresponding to equation 12, define the discrete two-time spatial propagator (relative deformation from step *k* to step *n*) as:

17 $$\begin{aligned} \boldsymbol{\Phi }(n,k) \;\equiv \; \textbf{F}_{\textrm{inc},n}\,\textbf{F}_{\textrm{inc},n-1}\cdots \textbf{F}_{\textrm{inc},k+1}, \qquad \boldsymbol{\Phi }(k,k)=\textbf{I}, \qquad \boldsymbol{\Phi }(n,k)=\textbf{F}_c(n)\,\textbf{F}_c(k)^{-1}. \end{aligned}$$

With $\boldsymbol{\Sigma }_0 = 0$, independence of the random steps yields on iterating (details in Supplementary Materials):

18 $$\begin{aligned} \boldsymbol{\Sigma }_n = \sum _{k=1}^n \boldsymbol{\Phi }(n,k) \, \Big [\ell ^2\textbf{I}\Big ]\, \boldsymbol{\Phi }(n,k)^{\!\top } = \sum _{k=1}^{n} \Big [\textbf{F}_c(n)\,\textbf{F}_c(k)^{-1}\Big ]\, \Big [\ell ^2\textbf{I}\Big ]\, \Big [\textbf{F}_c(k)^{-\!\top }\,\textbf{F}_c(n)^{\!\top }\Big ] \end{aligned}$$

from which

19 $$\begin{aligned} \textbf{D}_{\textrm{lab}}(t_n) = \frac{\ell ^2}{2t_n} \textbf{F}_n \left[ \sum _{k=1}^n \textbf{F}_k^{-1}\textbf{F}_k^{-\!\top }\right] \textbf{F}_n^{\!\top } \end{aligned}$$

where we write **F**_n_ for **F**_c_(n) and similarly **F**_k_ for short. Since $\textbf{X}(t) = \textbf{F}^{-1}(t)\,\textbf{y}(t)$, the term in brackets is related to the discrete time average of the particle diffusion in the reference frame. Substituting the diffusion scaling $\ell ^2=2D_0\Delta t$ and with $t_n=n\Delta t$

20 $$\begin{aligned} \textbf{D}_{\textrm{material}}(t_n) = \frac{D_0}{t_n}\! \sum _{k=1}^n \Delta t\,\textbf{F}_k^{-1}\textbf{F}_k^{-{\!\top }} \end{aligned}$$

This expression for the time-averaged reference frame diffusion $\textbf{D}_{\textrm{ref}}(t_n)$ is termed the *pull back* of the spatial diffusion to the reference frame. Similarly the diffusion measured in the spatial frame can be expressed as:

21 $$\begin{aligned} \textbf{D}_{\textrm{lab}}(t_n) =\textbf{F}_n\,\textbf{D}_{\textrm{material}}(t_n)\,\textbf{F}_n^{\!\top }. \end{aligned}$$

As $\Delta t\!\rightarrow \!0,\, n \rightarrow \infty$ we can replace the discrete sum in Eqs. (19), (20) by an integral, $\textbf{F}_n\!\rightarrow \!\textbf{F}(t)$, yielding

22 $$\begin{aligned} \textbf{D}_{\textrm{material}}(t) = {D_0}\left( \frac{1}{t}\!\int _0^t \textbf{F}^{-1}(s)\textbf{F}^{-{\!\top }}(s)\,ds\right) , \end{aligned}$$

and thus

23 $$\begin{aligned} \textbf{D}_{\textrm{lab}}(t) = \textbf{F}(t)\textbf{D}_{\textrm{material}}(t)\textbf{F}(t)^{{\!\top }} \end{aligned}$$

For a closed cycle where *t = T* is a complete period, $\textbf{F}(T)=\textbf{I}$, the laboratory measured diffusion at the end of the cycle equals the time-averaged material diffusion:

24 $$\begin{aligned} \textbf{D}_{\textrm{lab}}(T) = {D_0}\left( \frac{1}{t}\!\int _0^t \textbf{F}^{-1}(s)\textbf{F}^{-{\!\top }}(s)\,ds\right) = \textbf{D}_{\textrm{material}}(T). \end{aligned}$$

From the polar decomposition, $(\textbf{F} = \textbf{R} \cdot \textbf{U})$ in the case of isotropic diffusion, or when stretch is applied along the principal axes of the diffusion tensor (i.e., when the rotation tensor $\textbf{R} = \textbf{I}$ and so $\textbf{F} = \textbf{U}$), the reference diffusion is more easily calculated as:

25 $$\begin{aligned} \textbf{D}_{\textrm{lab}}(T) = \frac{D_0}{T}\int _0^T\textbf{U}^{-1}(s)\textbf{U}^{-1}(s)\,ds = \textbf{D}_{\textrm{material}}(T). \end{aligned}$$

This simplified form aligns with the equation provided by Reese et al.^35,39^, which they derived from the Stejskal-Tanner diffusion weighted imaging (DWI) signal equation^51^. However whereas equation 24^35,39^ is applicable only at the end of a cycle, equations 22 and 23 together provides a means to evaluate diffusion in the laboratory frame $\textbf{D}_{\textrm{lab}}$ at any time.

### Computational implementation of MCRW algorithms in deformed media

To account for strain in the MCRW algorithm, rather than applying strain at each diffusion step as in Equation 15, it is computationally efficient to employ two different temporal scales: a short timescale for diffusion ($\Delta t_D$) and a longer one for strain ($\Delta t_\epsilon$). The geometry (defined by a polygonal representation of the boundaries as in^4,30^) is thus updated by the deformation after $m = \Delta t_{\epsilon }/\Delta t_D$ diffusion steps. Thus equation 15 can be modified so

$$\boldsymbol{y}(t+\Delta t_{\varepsilon }) = \textbf{F}_{\textrm{inc},t+\Delta t_{\varepsilon }}\boldsymbol{y}(t) + \sum _{i=1}^m \boldsymbol{\eta }_{i},$$

the accuracy of which for comparatively large ratios is validated in Supplementary Materials.

#### Modelling semi-permeable barriers

The flux across barriers is determined by the following boundary condition:

26 $$\begin{aligned} \boldsymbol{j} \cdot \textbf{n} = \kappa \left( c^+ - c^- \right) , \end{aligned}$$

where $\boldsymbol{j}$ represents the diffusive flux, $\textbf{n}$ is the unit normal vector to the barrier surface, *κ* denotes the barrier permeability, and $c^+$ and $c^-$ are the property concentrations on either side of the barrier. Through flux continuity principles, the permeability *κ* relates to the diffusivity coefficient $D_b$ normal to the surface and the barrier thickness $\delta _s$ by:

27 $$\begin{aligned} \kappa = \frac{D_b}{\delta _s}. \end{aligned}$$

As the barrier permeability approaches infinity ($\kappa \rightarrow \infty$), the concentration discontinuity vanishes, and the barrier effectively disappears. Conversely, as the barrier permeability approaches zero ($\kappa \rightarrow 0$), the concentration discontinuity becomes large, and the barrier acts as an impermeable wall.

In the MCRW algorithm, Equation 26 is enforced by imposing a transit model that calculates a probability of transit when a particle encounters an intersection. The transit probabilities are based on the properties of the membrane and media either side of the membrane. The transit model aims to accurately represent the boundary condition such that it relates the diffusive flux to the jump in particle density $\Delta c=c^+ - c^-$ across the semi-permeable membrane. In this work, we assume the same diffusivity either side of the boundaries, which reduces the complexity of the transit model and has been widely studied^4–6^. The probability of crossing is then determined by:

28 $$\begin{aligned} p_t = \frac{2\kappa \delta _b}{D + 2\kappa \delta _b}, \end{aligned}$$

where $\delta _b$ is the distance from the walker position to the point of intersection with the membrane.

### Analytic evaluation of random walk for $\kappa \rightarrow \infty$ for cycles 1 and 2

We illustrate the analytic evaluation of the apparent diffusivity in an unbounded domain (equivalent to $\kappa \rightarrow \infty$) for the compression cycles 1 and 2, defined in Table 2. This serves as a validation of the MCRW result for infinite permeability shown in the results section. In these cycles, strain along the *z*-axis (either compressive or expansive) occurs from *t=0* to *t=T/2*, followed by the converse strain from *t=T/2* to *t=T*, where *T* corresponds to 2*n* timesteps. During the first phase, the *z* coordinate of points at time step *m* is rescaled as

$$z_m = z_0\left( 1+\varepsilon _{zz} {m}/{n}\right) ,$$

where for example in cycle 1, the strain in the first phase, $\varepsilon _{zz} = -0.18$. The incremental change in *z-*coordinate for either cycle can be expressed generally as

29 $$\begin{aligned} z_{m+1} = z_{m}\left[ 1 + \frac{\varepsilon _{zz}/n}{1+m\varepsilon _{zz}/n}\right] \end{aligned}$$

Corresponding expressions for $x_{m+1},y_{m+1}$ follow from the incompressibility constraint. For the relatively small strains considered, the varying incremental compression sequence of eq. 29 is well-approximated by a uniform sequence,

30 $$\begin{aligned} \textbf{F}_{\textrm{inc}}(a_i) = \operatorname {diag}(\alpha _i^{-1/2},\,\alpha _i^{-1/2}\, ,\alpha _i), \qquad \alpha _i = 1 + a_i\,\Delta t. \end{aligned}$$

such that during the first phase (compression) $a_i=a_1$ for *j=1,… ,n*, followed by the expansion where $a_i=a_2$ for *j=n+1,… ,2n*. As shown in Supplementary Materials, the cumulative effect of repeated identical deformations constitutes a geometric progression that is easily summed, leading to anisotropic diffusivities:

$$\frac{D_{zz}(T)}{D_0} =\frac{1}{2n}\!\left[ \alpha _2^{2n}G_n(\alpha _1^{2}) +G_n(\alpha _2^{2})\right] , \qquad \frac{D_{xx}(T)}{D_0} = \frac{D_{yy}(T)}{D_0} =\frac{1}{2n}\!\left[ \alpha _2^{-n}G_n(\alpha _1^{-1}) +G_n(\alpha _2^{-1})\right] ,$$

with $D_0=\ell ^2/(2\Delta t)$ as before and where $G_n(r)=(1-r^n)/(1-r)$.

As the process is cyclic, these diffusivities are identical in laboratory and material frames. In the limiting case of ${\Delta t \rightarrow 0}$, and considering a symmetric compression-expansion sequence (cycle 1 in Table 2), so $a_1 = -a$ , $a_2 = +a$, it can be shown (see Supplementary Materials) that

31 $$\begin{aligned} D_{zz}(T) = {D_0} \left( \frac{e^{a T}-1}{aT}\right) \;,\quad \;D_{xx}(T) = D_{yy}(T) = {D_0}\,\left( \frac{2\big (1 - e^{-a T/2}\big )}{aT}\right) \end{aligned}$$

*Numerical evaluation* To compare with the MCRW result (Fig. 3, $\kappa = \infty$), set $(1-a\Delta t)^n = 0.82$ corresponding to an 18% compression and $(1+a\Delta t)^n = 1/0.82$ as a restoring expansion. In the limiting case $\Delta t \rightarrow 0$ we find $aT = 2\ln {0.82} \approx 0.3969$ leading to:

(i) at the end of the complete cycle, substituting into equation 31 we obtain $D_{zz}(T) = 1.22{D_0},\,\,D_{xx} = D_{yy} = 0.91{D_0}$. This agrees with the computational result usng large *n* and small *Δ t* to two decimal places, whether in the latter case the computation applies an exact linear compression eq. 29 (which by evaluating Equation 24 gives $D_{zz}(T) = 1/(1+\epsilon _1) = 1/0.82$ or the approximate eq. 30).

(ii) an important distinction between cyclic and non-cyclic strain. This follows by considering the apparent diffusive spread at the end of the first half-cycle, using equation 23 with *t=T/2*; however now $D_{\textrm{lab}}(T/2) \ne D_{\textrm{material}}(T/2)$. In this case simulation of diffusive spread in the lab frame produces a very different result from that determined in the material frame, leading to:

32 $$\begin{aligned} D_{\textrm{lab},\,zz}=\big (e^{a_1 T/2}\big )^2\,\frac{1-e^{-a_1 T}}{a_1 T} = \frac{e^{a_1 T}-1}{a_1 T}{D_0} \qquad D_{\textrm{lab},\,xx}(T/2)=D_{\textrm{lab},\,yy}=\big (e^{-a_1 T/4}\big )^2\,\frac{2\big (e^{a_1 T/2}-1\big )}{a_1 T}{D_0}. \end{aligned}$$

Substituting $a_1T = -0.3969$ yields $D_{\textrm{lab},\,zz} = 0.8253{D_0}$ ; $D_{\textrm{lab},\,xx} = D_{\textrm{lab},\,yy} = 1.106{D_0}$, i.e. a reverse in the direction of strain effects observed at the end of the cycle.

## Supplementary Information

Supplementary Information.

## Data Availability

All data generated or analysed during this study are included in this published article and in the supplementary information file.
