# Supplementary material for: Diffusion in deforming semi-permeable media: a Monte Carlo random walk approach
Source: Sci Rep. 2026 Aug 7;16:36951. doi: 10.1038/s41598-026-64054-1 (PMC13736703; doi:10.1038/s41598-026-64054-1)
Supplement: Supplementary file 1 [file 41598_2026_64054_MOESM1_ESM.pdf]

Supplementary Information:
Diffusion in deforming semi-permeable media: a Monte Carlo random walk approach

Ignasi Alemany, Andrew Scott, Denis Doorly.

This section provides further details of the mathematical and computational analysis including results that show computational efficiency can be improved by decoupling the random walk and dynamic geometry updates.

1 Random walk model of diffusion during strain: unbounded medium

For simplicity we consider the intrinsic diffusivity of the material to be isotropic, but the method applies also to non-isotropic but homogeneous materials. The approach described can be applied to compare with the computational solutions for the simple case of infinite permeability. Specifically we set out to determine the effects of a globally affine deformation on the apparent diffusion tensor in a material.

1.1 Effect of material deformation on the apparent diffusion

Since a discrete sequence of infinitesimal increments can approximate a smooth deformation history, the effect of deformation on the mapping of a particle can be written as:

$$\boldsymbol{y}_n \; = \; \mathbf{F}_c(n)\,\mathbf{X}_n, \qquad \mathbf{F}_c(n) \; \equiv \; \mathbf{F}_{\text{inc},n}\,\mathbf{F}_{\text{inc},n-1}\cdots\mathbf{F}_{\text{inc},1}. \tag{1}$$

where $\boldsymbol{y}$ refers to laboratory coordinates and $\mathbf{X}$ to material coordinates. To incorporate the effect of diffusion using the MCRW approach, the position in the lab frame of each point $\boldsymbol{y}$ in a cloud of particles is updated from time step $j \to j{+}1$ through:

- a random isotropic displacement $\boldsymbol{\eta}_{j+1}$ with variance ℓ^2;
- an incremental deformation $\mathbf{F}_{\text{inc},j+1}$

Therefore the position of each particle in a cloud of particles evolves in the lab frame as:

$$\boldsymbol{y}_{j+1} = \mathbf{F}_{\text{inc},j+1}\boldsymbol{y}_j + \boldsymbol{\eta}_{j+1}, \qquad \text{where} \qquad \mathbb{E}[\boldsymbol{\eta}_{j+1}\boldsymbol{\eta}_{j+1}^\top] = \ell^2\mathbf{I}, \tag{2}$$

This formulation captures both the effective advection of the particles and the alteration in apparent diffusivity through the cumulative operation of the incremental deformation on the random steps. The spatial covariance in the lab frame $\boldsymbol{\Sigma}_j = \mathbb{E}[\boldsymbol{y}_j\boldsymbol{y}_j^\top]$ evolves at each step as:

$$\boldsymbol{\Sigma}_{j+1} = \mathbf{F}_{\text{inc},j+1}\boldsymbol{\Sigma}_j\mathbf{F}_{\text{inc},j+1}^\top + \ell^2\mathbf{I}. \tag{3}$$

Corresponding to equation 2, define the discrete two-time spatial propagator (relative deformation from step k to step n) as:

$$\boldsymbol{\Phi}(n,k) \; \equiv \; \mathbf{F}_{\text{inc},n}\,\mathbf{F}_{\text{inc},n-1}\cdots\mathbf{F}_{\text{inc},k+1}, \qquad \boldsymbol{\Phi}(k,k) = \mathbf{I}, \qquad \boldsymbol{\Phi}(n,k) = \mathbf{F}_c(n)\,\mathbf{F}_c(k)^{-1}. \tag{4}$$

Considering the first few steps in updating the position of a particle leads to the expression for n updates, namely

$$
\begin{aligned}
\boldsymbol{y}_0 &= \boldsymbol{y}_0, \\
\boldsymbol{y}_1 &= \mathbf{F}_{\text{inc},1}\,\boldsymbol{y}_0 + \boldsymbol{\eta}_1, \\
\boldsymbol{y}_2 &= \mathbf{F}_{\text{inc},2}\,\boldsymbol{y}_1 + \boldsymbol{\eta}_2 = \underbrace{\mathbf{F}_{\text{inc},2}\mathbf{F}_{\text{inc},1}}_{\boldsymbol{\Phi}(2,0)}\,\boldsymbol{y}_0 + \underbrace{\mathbf{F}_{\text{inc},2}}_{\boldsymbol{\Phi}(2,1)}\,\boldsymbol{\eta}_1 + \boldsymbol{\eta}_2, \\
\boldsymbol{y}_3 &= \mathbf{F}_{\text{inc},3}\,\boldsymbol{y}_2 + \boldsymbol{\eta}_3 = \underbrace{\mathbf{F}_{\text{inc},3}\mathbf{F}_{\text{inc},2}\mathbf{F}_{\text{inc},1}}_{\boldsymbol{\Phi}(3,0)}\,\boldsymbol{y}_0 + \underbrace{\mathbf{F}_{\text{inc},3}\mathbf{F}_{\text{inc},2}}_{\boldsymbol{\Phi}(3,1)}\,\boldsymbol{\eta}_1 + \underbrace{\mathbf{F}_{\text{inc},3}}_{\boldsymbol{\Phi}(3,2)}\,\boldsymbol{\eta}_2 + \boldsymbol{\eta}_3 \\
&\;\;\vdots \\
\boldsymbol{y}_n &= \mathbf{F}_{\text{inc},n}\,\boldsymbol{y}_{n-1} + \boldsymbol{\eta}_n = \boldsymbol{\Phi}(n,0)\,\boldsymbol{y}_0 + \sum_{j=0}^{n-1}\boldsymbol{\Phi}(n,j+1)\,\boldsymbol{\eta}_{j+1}.
\end{aligned} \tag{5}
$$

So with $\boldsymbol{\Sigma}_0 = 0$, independence of the steps yields on iterating

$$
\mathbb{E}[\boldsymbol{y}_n\boldsymbol{y}_n^\top] = \boldsymbol{\Sigma}_n = \sum_{k=1}^{n}\boldsymbol{\Phi}(n,k)\left[\ell^2\mathbf{I}\right]\boldsymbol{\Phi}(n,k)^\top = \sum_{k=1}^{n}\left[\mathbf{F}_c(n)\,\mathbf{F}_c(k)^{-1}\right]\left[\ell^2\mathbf{I}\right]\left[\mathbf{F}_c(k)^{-\top}\,\mathbf{F}_c(n)^\top\right] \tag{6}
$$

Thus, the diffusivity, as it appears in the lab frame, at $t_n = n\delta t$ is

$$
\mathbf{D}_{\text{lab}}(t_n) = \frac{\boldsymbol{\Sigma}_n}{2t_n} = \frac{\ell^2}{2t_n}\sum_{k=1}^{n}\boldsymbol{\Phi}(n,k)\boldsymbol{\Phi}(n,k)^\top. \tag{7}
$$

Write $\mathbf{F}_c(n)$ as $\mathbf{F}_n$ for short and similarly $\mathbf{F}_k$ for $\mathbf{F}_k(n)$. Substituting $\boldsymbol{\Phi}(n,k) = \mathbf{F}_n\mathbf{F}_k^{-1}$ into (7) above yields the relation:

$$
\begin{aligned}
\mathbf{D}_{\text{lab}}(t_n) &= \frac{\ell^2}{2t_n}\sum_{k=1}^{n}\mathbf{F}_n\mathbf{F}_k^{-1}(\mathbf{F}_k^{-1})^\top\mathbf{F}_n^\top \\
&= \frac{\ell^2}{2t_n}\,\mathbf{F}_n\left[\sum_{k=1}^{n}\mathbf{F}_k^{-1}\mathbf{F}_k^{-T}\right]\mathbf{F}_n^\top.
\end{aligned} \tag{8}
$$

Since $\mathbf{X}(t) = \mathbf{F}^{-1}(t)\,\mathbf{y}(t)$, or equivalently $\mathbf{X}_k = \mathbf{F}_k^{-1}\,\boldsymbol{y}_k$, the corresponding step update in the material frame is

$$
\mathbf{X}_{k+1} = \mathbf{F}_{k+1}^{-1}\,\boldsymbol{y}_{k+1} = \mathbf{F}_{k+1}^{-1}\Big(\mathbf{F}_{\text{inc},k+1}\,\boldsymbol{y}_k + \boldsymbol{\eta}_{k+1}\Big) = \mathbf{X}_k + \mathbf{F}_{k+1}^{-1}\,\boldsymbol{\eta}_{k+1} \tag{9}
$$

so that the random increments in the material frame have covariance $\mathbf{F}_{k+1}^{-1}\,\mathbb{E}[\boldsymbol{\eta}_{k+1}\boldsymbol{\eta}_{k+1}^\top]\,\mathbf{F}_{k+1}^{-\top} = \ell^2\,\mathbf{F}_{k+1}^{-1}\mathbf{F}_{k+1}^{-\top}$.

Thus the term in brackets in Equation (8) corresponds to the discrete time average of the particle diffusion in the material frame. Substituting the diffusion scaling $\ell^2 = 2D_0\delta t$ and with $t_n = n\delta t$

$$
\mathbf{D}_{\text{material}}(t_n) = \frac{D_0}{t_n}\sum_{k=1}^{n}\delta t\,\mathbf{F}_k^{-1}\mathbf{F}_k^{-T} \tag{10}
$$

This expression $\mathbf{D}_{\text{material}}(t_n)$ is termed the *pull back* of the lab (or spatial) diffusion to the material (reference) frame. Similarly the diffusion measured in the laboratory frame $\mathbf{D}_{\text{lab}}$ (which could also be termed the apparent diffusion $\mathbf{D}_{\text{app}}$), can be expressed as:

$$
\mathbf{D}_{\text{lab}}(t_n) = \mathbf{F}_n\,\mathbf{D}_{\text{material}}(t_n)\,\mathbf{F}_n^\top. \tag{11}
$$

As $\delta t \to 0$, we can replace the discrete sum by a Riemann integral, $\mathbf{F}_n \to \mathbf{F}(t)$, yielding

$$
\mathbf{D}_{\text{lab}}(t) = \frac{D_0}{t}\int_0^t \boldsymbol{\Phi}(t,s)\boldsymbol{\Phi}(t,s)^\top\,ds = \frac{D_0}{t}\int_0^t \mathbf{F}(t)\mathbf{F}(\mathbf{s})^{-1}\mathbf{F}(s)^{-T}\mathbf{F}(t)^T\,ds \tag{12}
$$

With

$$\mathbf{D}_{\text{material}}(t) = \frac{D_0}{t}\int_0^t \mathbf{F}^{-1}(s)\mathbf{F}^{-T}(s)\,ds, \tag{13}$$

the continuous analog of equation 11 is

$$\mathbf{D}_{\text{lab}}(t) = \mathbf{F}(t)\mathbf{D}_{\text{material}}(t)\mathbf{F}(t)^T \tag{14}$$

For a closed cycle where $t = T$ is a complete period, $\mathbf{F}(T) = \mathbf{I}$, the spatial or apparent diffusion at the end of the cycle equals the time-averaged pull-back or reference frame diffusion:

$$\mathbf{D}_{\text{lab}}(T) = \frac{D_0}{T}\int_0^T \mathbf{F}^{-1}(s)\mathbf{F}^{-T}(s)\,ds = \mathbf{D}_{\text{material}}(T). \tag{15}$$

From the polar decomposition, ($\mathbf{F} = \mathbf{R}\cdot\mathbf{U}$) in the case of isotropic diffusion, or when stretch is applied along the principal axes of the diffusion tensor (i.e., when the rotation tensor $\mathbf{R} = \mathbf{I}$ and so $\mathbf{F} = \mathbf{U}$), the end cycle apparent diffusion coefficient simplifies to:

$$\mathbf{D}_{\text{lab}}(T) = \frac{D_0}{T}\int_0^T \mathbf{U}^{-1}(s)\mathbf{U}^{-1}(s)\,ds = \mathbf{D}_{\text{material}}(T). \tag{16}$$

This simplified form aligns with the equation provided by Reese et al. [1, 2], which they derived from the Stejskal-Tanner diffusion weighted imaging (DWI) signal equation[3]. However whereas equation 16[1, 2] is applicable only at the end of a cycle, equation 12 provides a means to evaluate $\mathbf{D}_{\text{app}}$ at any time. In the following section we show how it is possible to compute exactly the random walk result for apparent diffusivity for an isotropic medium and present the limiting expressions ($n \to \infty$, $\delta t \to 0$, $2n\delta t = T$)

1.2 Effects of deformation on strain cycles 1 and 2

Here we show how the theory developed above can be used to derive expressions for the effect of strain in an unbounded medium for the linear compression and expansion cycles 1 and 2, including where the strain does not complete a cycle. Whereas equation 16 above is applicable only at the end of a cycle, equation 12 provides a means to evaluate $\mathbf{D}_{\text{lab}}$ at any time. As described in the paper, we take the example of an isotropic material subject to a linear-in-time uniaxial and incompressible compression along the z axis, followed by a compensating expansion back to its original dimensions. Though not exact, a good approximation to constant strain for relatively small total strain is a constant incremental strain; the advantage of the latter is it allows summation as a simple geometric series as shown here.

Considering cycle 1 or cycle 2, let respectively compression (cycle 1) or expansion (cycle 2) occur from $t = 0$ to $t = T/2$, followed by the converse (respectively expansion or compression) occur from $t = T/2$ to $t = T$ and where T corresponds to $2N$ timesteps. Each step applies an incompressible uniaxial stretch or compression:

$$\mathbf{F}_{\text{inc}}(a_i) = \text{diag}(\alpha_i^{-1/2},\, \alpha_i^{-1/2},\, \alpha_i), \qquad \alpha_i = 1 + a_i\,\delta t.$$

such that during the first phase (compression) $a_i = a_1$ for $j = 1, \dots, N$, followed by the expansion where $a_i = a_2$ for $j = N+1, \dots, 2N$. Because these increments are diagonal, they commute:

$$\mathbf{\Phi}(n,k) = \text{diag}\left(\prod_{j=k+1}^{n} \alpha_j^{-1/2},\ \prod_{j=k+1}^{n} \alpha_j^{-1/2},\ \prod_{j=k+1}^{n} \alpha_j\right).$$

To illustrate we take the apparent diffusion observed at two points.

(i) At mid-cycle: ($n = N$, end of first phase i.e. compression for cycle 1, expansion for cycle 2).
All steps are of the same magnitude in each phase ($\alpha_j = \alpha_1$), so

$$\Phi(N,k) = \text{diag}(\alpha_1^{-m/2}, \alpha_1^{-m/2}, \alpha_1^{m}), \quad m = N - k.$$

Summing over $k = 1, \dots, N$ (the sum of simple geometric progressions with multipliers $r = \alpha_1$ or $r = \alpha_1^{-1/2}$

$$\Sigma_{zz}(T/2) = \ell^2 G_N(\alpha_1^2), \quad \Sigma_{xx}(T/2) = \Sigma_{yy}(T/2) = \ell^2 G_N(\alpha_1^{-1}),$$

where $G_N(r) = (1 - r^N)/(1 - r)$

(ii) At end-cycle: ($n = 2N$, the strains reverse, (e.g. for cycle 1 after the compression in phase 1 comes expansion). So the total increments comprise two parts:

- the sum of all the steps during the first phase multiplied by $(\alpha_2^2)^N$ (i.e multiplied by all N steps during the second phase);
- added to this is the sum of the N steps in phase 2.

Thus

$$\Sigma_{zz}(T) = \ell^2 \left[\alpha_2^{2N} G_N(\alpha_1^2) + G_N(\alpha_2^2)\right] \quad \Sigma_{xx}(T) = \Sigma_{yy}(T) = \ell^2 \left[\alpha_2^{-N} G_N(\alpha_1^{-1}) + G_N(\alpha_2^{-1})\right]$$

Therefore

$$\frac{D_{zz}(T)}{D_0} = \frac{1}{2N}\left[\alpha_2^{2N} G_N(\alpha_1^2) + G_N(\alpha_2^2)\right], \qquad \frac{D_{xx}(T)}{D_0} = \frac{D_{yy}(T)}{D_0} = \frac{1}{2N}\left[\alpha_2^{-N} G_N(\alpha_1^{-1}) + G_N(\alpha_2^{-1})\right], \quad (17)$$

with $D_0 = \ell^2/(2\delta t)$ as before. In the limiting case of $\delta t \to 0; N \to \infty \,|\, 2N\delta t = T$, from the definition of the exponential

$$G_N(\alpha_i^2) = \frac{1 - (1 + a_i\,\delta t)^{2N}}{1 - (1 + a_i\,\delta t)^2} \quad \to \quad \frac{e^{\,2\,a_i\,N\delta t} - 1}{2\,a_i\,\delta t} \quad (18)$$

and similar expression for $G_N(\alpha_i^{-1})$. Thus substituting $T = 2N\delta t$, we obtain

$$D_{zz}(T) \;\to\; \frac{D_0}{T}\left[\frac{e^{a_2 T}\left(e^{a_1\,T} - 1\right)}{2\,a_1} + \frac{e^{a_2\,T} - 1}{2\,a_2}\right] \quad (19)$$

$$D_{xx}(T), D_{yy}(T) \to \frac{D_0}{T}\left[\frac{e^{-\,a_2\,T/2}\left(1 - e^{-\,a_1\,T/2}\right)}{a_1} + \frac{1 - e^{-\,a_2\,T/2}}{a_2}\right]. \quad (20)$$

Material frame analysis. In the equivalent analysis in the material frame, the coordinates are fixed, but the diffusivity value is rescaled by the contemporaneous strain α_i^n so the step variance in z becomes ℓ^2/α_i^{2n} and in x, y is $\ell^2\alpha_i^n$. Comparable analysis to the above then gives for phase 1 ($0 < t < T/2$):

$$\Sigma_{zz}^{(1),\mathrm{mat}}(T/2) = \ell^2 \sum_{n=0}^{N-1} \frac{1}{\alpha_1^{2n}} = \ell^2 G_N(\alpha_1^{-2}), \quad \Sigma_{xx}^{(1),\mathrm{mat}}(T/2) = \ell^2 \sum_{n=0}^{N-1} \alpha_1^n = \ell^2 G_N(\alpha_1). \quad (21)$$

For phase 2 ($T/2 < t < T$):

$$\Sigma_{zz}^{(2),\mathrm{mat}} = \ell^2 \sum_{n=0}^{N-1} \frac{1}{(\alpha_1^{2N}\alpha_2^{2n})} = \frac{\ell^2}{\alpha_1^{2N}} \sum_{n=0}^{N-1} \alpha_2^{-2j} = \frac{\ell^2}{\alpha_1^{2N}} G_N(\alpha_2^{-2}), \quad (22)$$

$$\Sigma_{xx}^{(2),\mathrm{mat}} = \ell^2 \sum_{n=0}^{N-1} \alpha_1^N \alpha_2^n = \ell^2 \alpha_1^N G_N(\alpha_2). \quad (23)$$

Summing both phases gives the alternative form:

$$D_{zz}^{\mathrm{mat}}(T) = \frac{D_0}{2N}\left[G_N(\alpha_1^{-2}) + \alpha_1^{-2N} G_N(\alpha_2^{-2})\right], \quad D_{xx,yy}^{\mathrm{mat}}(T) = \frac{D_0}{2N}\left[G_N(\alpha_1) + \alpha_1^N G_N(\alpha_2)\right]. \quad (24)$$

Applying the same limiting process as for the lab frame, we obtain

$$D_{zz}^{\mathrm{mat}}(T) \to \frac{D_0}{T}\left[\frac{1 - e^{-a_1 T}}{2a_1} + \frac{e^{-a_1 T}(1 - e^{-a_2 T})}{2a_2}\right], \quad D_{xx}^{\mathrm{mat}}(T) \to \frac{D_0}{T}\left[\frac{e^{a_1 T/2} - 1}{a_1} + \frac{e^{a_1 T/2}(e^{a_2 T/2} - 1)}{a_2}\right]. \quad (25)$$

Note the material form of the result is different from that in the lab frame as expected. But both formulations reduce to the same result for a cycle, with the final form for each of the linear cycles given below. First we compare with the continuum expressions.

Comparing with the continuum expressions, for the given deformation $\mathbf{F}$ with

$$\mathbf{F}^{-1}(s)\mathbf{F}^{-\top}(s) = \mathrm{diag}\big(\alpha(s),\, \alpha(s),\, \alpha(s)^{-2}\big), \tag{26}$$

where in the limiting form of the approximation to a linear strain as a series of constant increments,

$$\alpha(s) = \begin{cases} e^{\,a_1 s}, & 0 \le s \le T/2, \\ e^{\,a_1 T/2}\, e^{\,a_2 (s - T/2)}, & T/2 < s \le T. \end{cases} \tag{27}$$

Substituting into equation 15 with $\mathbf{F}(T) = 1$

$$\mathbf{D}_{\mathrm{lab}(T)} = \mathbf{D}_{\mathrm{material}(T)} = \frac{D_0}{T} \int_0^T \big(\mathbf{F}^{-1}(s)\mathbf{F}^{-\top}(s)\big)\, ds. \tag{28}$$

we obtain:

$$D_{zz}(T) = \frac{D_0}{T}\left[\int_0^{T/2} e^{-2a_1 s}\, ds + e^{-2a_1 T/2} \int_{T/2}^{T} e^{-2a_2(s-T/2)}\, ds\right], \tag{29}$$

$$D_{xx}(T) = D_{yy}(T) = \frac{D_0}{T}\left[\int_0^{T/2} e^{\,a_1 s}\, ds + e^{\,a_1 T/2} \int_{T/2}^{T} e^{\,a_2(s-T/2)}\, ds\right], \tag{30}$$

Evaluating the integrals recoves the equations 25.

Simplified form for cycles.

For cycle 1: C1→C2→C1:
From Equation (19) set $a_1 = -a$, $a_2 = +a$ yielding

$$D_{zz}(T) = D_0\left(\frac{e^{aT} - 1}{aT}\right), \quad D_{xx}(T) = D_{yy}(T) = D_0\left(\frac{2\big(1 - e^{-aT/2}\big)}{aT}\right) \tag{31}$$

For cycle 2: C2→C1→C2:
setting $a_1 = a$; $a_2 = -a$ we obtain the converse:

$$D_{zz}(T) = D_0\left(\frac{1 - e^{-aT}}{aT}\right), \quad D_{xx}(T) = D_{yy}(T) = D_0\left(\frac{2\big(e^{-aT/2} - 1\big)}{aT}\right) \tag{32}$$

Exact calculation of effect of strain for cycle.

The exact expression for a linear strain has

$$\mathbf{F}(t) = 1 + \epsilon\frac{t}{T}$$

where ϵ is the total strain. Thus

$$D_{zz}(T) = \frac{D_0}{T}\left[\int_0^{T/2} \frac{1}{\big(1 + \epsilon_1 \frac{s}{T}\big)^2}\, ds + \int_{T/2}^{T} \frac{1}{(1+\epsilon_1)^2 (1 + \epsilon_2 \left(\frac{s - T/2}{T/2}\right)^2}\, ds\right], \tag{33}$$

with corresponding expressions for D_{xx}, D_{yy}.

Under the cyclic condition, $(1+\epsilon_1)(1+\epsilon_2) = 1$, we obtain

$$D_{zz}(T) = \frac{D_0}{(1+\epsilon_1)} \tag{34}$$

---

2 Diffusion in an impermeable cell

In this section we describe an analytic solution that can be used to validate the computational results for the impermeable cell under strain. For this reason we have extended the well-known solution for diffusion in an impermeable slab Crank [4] (equation 4.56) to the case of an impermeable 3-D rectangular sided cell under strain. It is then possible to compare our implementation of the random walk method in 3D applied to the cell elements of the whole domain.

2.1 Derivation of an expression for the MSD $\langle (X - X_0)^2 \rangle$

For analysis of a body under deformation, it is convenient to work in fixed material coordinates, as this reduces the equivalent advection-diffusion equation in lab coordinates to just diffusion, though with time-dependent diffusivity $\mathbf{D}_{material}(t)$. For a general time-varying uniaxial incompressible strain along the z-axis with length scaled by $h(t)$, the deformation gradient $\mathbf{F}$ has diagonal form

$$\mathbf{F} = diag(h(t)^{-\frac{1}{2}}, h(t)^{-\frac{1}{2}}, h(t)) \tag{35}$$

leading to the diffusion equation expressed in material coordinates as:

$$\frac{\partial \tilde{c}}{\partial t} = D_0 h(t) \frac{\partial^2 \tilde{c}}{\partial X^2} + D_0 h(t) \frac{\partial^2 \tilde{c}}{\partial Y^2} + \frac{D_0}{h(t)^2} \frac{\partial^2 \tilde{c}}{\partial Z^2}, \tag{36}$$

where $\tilde{c}$ represents concentration expressed in material coordinates. From the results derived in the main paper and above, the time-varying diffusivity

$$\mathbf{D}_{\text{material}} = diag(D_X(t), D_Y(t), D_Z(t)) = diag\left(D_0 h(t), D_0 h(t), \frac{D_0}{h(t)^2} \right) \tag{37}$$

reduces for a complete cycle of length T to

$$\mathbf{D}_{\text{material}}(T) = D_0 \left(\frac{1}{T} \int_0^T \mathbf{F}^{-1}(s) \mathbf{F}^{-T}(s)\, ds \right) = \frac{D_0}{T} \int_0^T diag\left(h(s), h(s), \frac{1}{h(s)^2} \right) ds \tag{38}$$

We now describe a solution for the MSD on the **fixed** domain $X \in [0, L_X]$, $Y \in [0, L_Y]$, $Z \in [0, L_Z]$, that is derived from the result in Crank (equation 4.56 in ref) for 1D diffusion on $X \in [0, L]$ with no-flux boundaries and initial condition $c_0(X)$:

$$c(X,t) = \sum_{m=0}^{\infty} A_m \cos\left(\frac{m\pi X}{L}\right) \exp\left(-\frac{D_0 m^2 \pi^2 t}{L^2}\right), \qquad A_m = \frac{\alpha_m}{L} \int_0^L c_0(X') \cos\left(\frac{m\pi X'}{L}\right) dX', \tag{39}$$

where $\alpha_0 = 1$ and $\alpha_m = 2$ for $m \geq 1$.
Substituting $c_0(X') = \delta(X' - X_0)$ gives the Green's function $G(X_0, X, t)$ (equivalent to the propagator for the pdf of X); the coefficients thus reduce to $A_m = (\alpha_m / L) \cos(m\pi X_0 / L)$, so that:

$$G(X_0, X, t) = \frac{1}{L} + \frac{2}{L} \sum_{m=1}^{\infty} \cos\left(\frac{m\pi X_0}{L}\right) \cos\left(\frac{m\pi X}{L}\right) \exp\left(-\frac{m^2 \pi^2 D_0 t}{L^2}\right). \tag{40}$$

Under a time varying deformation, the transformation to material coordinates multiplies the intrinsic diffusivity D_0 by a function of the strain, e.g. $D_0 h(t)$ for x or y directions from above. We can combine this into a **time-varying diffusivity** $D(t)$.

Then expanding G in the same cosine basis, now with time-dependent coefficients $A_m(X_0, t)$:

$$G(X_0, X, t) = \sum_{m=0}^{\infty} A_m(X_0, t) \cos\left(\frac{m\pi X}{L}\right). \tag{41}$$

Substituting into $\partial G / \partial t = D(t)\, \partial^2 G / \partial X^2$ and matching coefficients gives:

$$\frac{dA_m}{dt} = -\left(\frac{m\pi}{L}\right)^2 D(t)\, A_m, \tag{42}$$

from which:

$$A_m(X_0,t) = A_m(X_0,0)\exp\left(-\frac{m^2\pi^2 I_D(t)}{L^2}\right), \qquad I_D(t) = \int_0^t D(t')\,dt'. \tag{43}$$

Note for a cuboidal cell L is adjusted to l_x, l_y, l_z depending on the coordinate (X can stand for X, Y or Z). Correspondingly $I_D(t)$ also depends on the strain in the particular coordinate direction.
Substituting $G(X_0, X, 0) = \delta(X - X_0)$ yields the same coefficients $A_m = (\alpha_m/L)\cos(m\pi X_0/L)$ for the initial condition as in the above, namely

$$A_0(X_0,0) = \frac{1}{L}, \qquad A_m(X_0,0) = \frac{2}{L}\cos\left(\frac{m\pi X_0}{L}\right), \quad m \geq 1, \tag{44}$$

The extension to Crank's solution, allowing for time-varying diffusivity $D(t)$ (due to transformation to material coordinates) can thus be expressed as

$$G(X_0,X,t) = \frac{1}{L} + \frac{2}{L}\sum_{m=1}^{\infty}\cos\left(\frac{m\pi X_0}{L}\right)\cos\left(\frac{m\pi X}{L}\right)\exp\left(-\frac{m^2\pi^2 I_D(t)}{L^2}\right), \qquad I_D = \int_0^t D(t')\,dt' \tag{45}$$

which differs only in replacing $D_0 t$ with $I_D(t)$

With the geometry aligned with the coordinate axes, the diffusion tensor determined *at the end of a cycle* reduces to

$$\mathbf{D} = \frac{1}{2T} diag\left(\langle\Delta X^2\rangle, \langle\Delta Y^2\rangle, \langle\Delta Z^2\rangle\right) = \frac{1}{2T} diag\left(\langle\Delta x^2\rangle, \langle\Delta y^2\rangle, \langle\Delta z^2\rangle\right), \tag{46}$$

since material and lab coordinates are then identical.

Thus to evaluate $\mathbf{D}$, we require the mean square displacement (MSD) in each of the three coordinate directions. For a given start position X_0,

$$\langle (X - X_0)^2\rangle|_{X_0} = \int_0^L (X - X_0)^2\, G(X_0, X, t)\, dX.$$

Averaging over all possible starting positions X_0,

$$\langle (X - X_0)^2\rangle = \int_0^L \langle (X - X_0)^2\rangle|_{X_0} \cdot \frac{1}{L}\, dX_0 = \frac{1}{L}\int_0^L\int_0^L (X - X_0)^2\, G(X_0, X, t)\, dX\, dX_0. \tag{47}$$

Substituting for the propagator,

$$\begin{aligned}\langle (X - X_0)^2\rangle &= \frac{1}{L}\int_0^L\int_0^L (X - X_0)^2\left[\frac{1}{L} + \frac{2}{L}\sum_{m=1}^{\infty}\cos\left(\frac{m\pi X_0}{L}\right)\cos\left(\frac{m\pi X}{L}\right)e^{-m^2\pi^2 I_D(t)/L^2}\right]dX\,dX_0\\ &= \frac{1}{L^2}\int_0^L\int_0^L (X - X_0)^2\, dX\, dX_0\\ &\quad + \frac{2}{L^2}\sum_{m=1}^{\infty} e^{-m^2\pi^2 I_D(t)/L^2}\int_0^L\int_0^L (X - X_0)^2\cos\left(\frac{m\pi X_0}{L}\right)\cos\left(\frac{m\pi X}{L}\right)dX\,dX_0.\end{aligned} \tag{48}$$

The first term on the right hand side is just the variance of the difference of two uniform random variables on [0,L] which is $L^2/6$.
Expanding the integral in the second term,

$$\begin{aligned}&\int_0^L\int_0^L (X - X_0)^2\cos\left(\frac{m\pi X_0}{L}\right)\cos\left(\frac{m\pi X}{L}\right)dX\,dX_0\\ &\qquad = \int_0^L X^2\cos\left(\frac{m\pi X}{L}\right)dX\int_0^L\cos\left(\frac{m\pi X_0}{L}\right)dX_0\\ &\qquad + \int_0^L\cos\left(\frac{m\pi X}{L}\right)dX\int_0^L X_0^2\cos\left(\frac{m\pi X_0}{L}\right)dX_0\\ &\qquad - 2\int_0^L X\cos\left(\frac{m\pi X}{L}\right)dX\int_0^L X_0\cos\left(\frac{m\pi X_0}{L}\right)dX_0\end{aligned} \tag{49}$$

The first two terms on the right hand side of the above equation are zero, since each has a multiplying cosine integral that yields $(L/m\pi)\sin(m\pi X/L)\Big|_0^L = 0$. The last term can be written as the product I_m^2 of definite integrals, where

$$I_m = \int_0^L X\cos\left(\frac{m\pi X}{L}\right)dX. \tag{50}$$

Integrating by parts reduces I_m to

$$I_m = -\int_0^L \frac{L}{m\pi}\sin\left(\frac{m\pi X}{L}\right)dX = \frac{L^2}{(m\pi)^2}\left[(-1)^m - 1\right].$$

For m even, $I_m = 0$; for m odd, $I_m = -\dfrac{2L^2}{(m\pi)^2}$ Hence

$$I_m^2 = \begin{cases} 4L^4/(m\pi)^4 & m \text{ odd,} \\ 0 & m \text{ even.} \end{cases} \tag{51}$$

So gathering terms,

$$\begin{aligned} \langle (X - X_0)^2 \rangle &= \frac{L^2}{6} + \frac{2}{L^2}\sum_{m=1}^{\infty} \exp\big(-m^2\pi^2 I_D(t)/L^2\big)(-2I_m^2) \\ &= \frac{L^2}{6} + \frac{2}{L^2}\sum_{m=1}^{\infty} \exp\big(-m^2\pi^2 I_D(t)/L^2\big)(-2)\cdot 4L^4/(m\pi)^4, \quad m \text{ odd,} \end{aligned} \tag{52}$$

Writing $m = 2n - 1$ for $n = 1, 2, 3, \ldots$,

$$\langle (X - X_0)^2 \rangle = \frac{L^2}{6} + \frac{2}{L^2}\sum_{n=1}^{\infty} \exp\big(-(2n-1)^2\pi^2 I_D(t)/L^2\big)\cdot(-2)\cdot\frac{4L^4}{(2n-1)^4\pi^4}$$

Finally

$$\langle (X - X_0)^2 \rangle = L^2\left[\frac{1}{6} - \frac{16}{\pi^4}\sum_{n=1}^{\infty}\frac{1}{(2n-1)^4}\exp\left(-\frac{(2n-1)^2\pi^2 I_D(t)}{L^2}\right)\right];\ I_D = \int_0^t D(t')\,dt' \tag{53}$$

More generally,

$$\langle (X_i - X_{0,i})^2 \rangle = L_i^2\left[\frac{1}{6} - \frac{16}{\pi^4}\sum_{n=1}^{\infty}\frac{1}{(2n-1)^4}\exp\left(-\frac{(2n-1)^2\pi^2 I_D(t)}{L_i^2}\right)\right];\ I_{D,i} = \int_0^t D_i(t')\,dt' \tag{54}$$

where i represents X, Y or Z.

As a check, in the limit $L \to \infty$, the exponential term becomes small.

Then let $\delta_n = \dfrac{(2n-1)^2\pi^2\, I_{D,i}}{L_i^2}$, and expanding to second order:

$$\exp(-\delta_n) = 1 - \delta_n + \frac{\delta_n^2}{2} - \cdots \tag{55}$$

Substituting into the MSD:

$$\langle (\Delta X_i)^2 \rangle = L_i^2\left[\frac{1}{6} - \frac{16}{\pi^4}\sum_{n=1}^{\infty}\frac{1}{(2n-1)^4}\left(1 - \delta_n + \frac{\delta_n^2}{2} - \cdots\right)\right]. \tag{56}$$

Since $\displaystyle\sum_{n=1}^{\infty}\frac{1}{(2n-1)^4} = \frac{\pi^4}{96}$, the first two terms cancel;

the next term inside the the curved brackets is $\delta_n = \dfrac{(2n-1)^2\pi^2 I_{D,i}}{L_i^2}$:

Substituting for δ_n and using

$$\sum_{n=1}^{\infty}\frac{1}{(2n-1)^2} = \frac{\pi^2}{8},$$

$$\begin{aligned}\langle(\Delta X_i)^2\rangle &= L_i^2\left[\frac{16}{\pi^4}\sum_{n=1}^{\infty}\frac{1}{(2n-1)^4}\frac{(2n-1)^2\pi^2\, I_{D,i}}{L_i^2} - \mathcal{O}\left(\frac{1}{L_i^4}\right)\right] \\ &= \rightarrow 2I_{D,i} \end{aligned} \tag{57}$$

2.2 Evaluation of $\langle(X_i - X_{0,i})^2\rangle$ for $i = Z$, i.e. $\langle\Delta Z^2\rangle$ for cycle 1

To illustrate evaluation of eq. (54), consider cycle 1 (from the main body of the paper), defined by $\mathbf{F} = diag(h(t)^{-1/2}, h(t)^{-1/2}, h(t))$ where

$$h(t) = \begin{cases} 1 + \dfrac{2a_1 t}{T} & 0 \le t \le T/2, \\ (1+a_1)\left(1 + \dfrac{2a_2(t - T/2)}{T}\right) & T/2 \le t \le T, \end{cases} \tag{58}$$

$a_1T/2$ = -0.18; $a_2T/2$ = +0.18.

For the Z component, $I_{D,Z}(t) = D_0 \int_0^t \dfrac{ds}{h^2(s)}$, so at cycle end $t = T$,

$$\begin{aligned} I_{D,Z}(T) &= \int_0^T D_0 \frac{ds}{h(s)^2} \\ &= D_0\left[\int_0^{T/2}\frac{ds}{\left(1+\dfrac{2a_1 s}{T}\right)^2} + \int_{T/2}^{t}\frac{ds}{(1+a_1)^2\left(1+\dfrac{2a_2(s-T/2)}{T}\right)^2}\right] \\ &= D_0\left[\frac{T}{2(1+a_1)} + \frac{T}{2(1+a_1)^2(1+a_2)}\right] \\ &= D_0\frac{T}{1+a_1} \end{aligned} \tag{59}$$

Thus for cycle 1,

$$I_{D,Z}(T) = D_0\frac{1000}{0.82} = 1219.5D_0 \ \ (\mathrm{ms}\cdot\mu\mathrm{m}^2/\mathrm{ms})$$

Starting from the full MSD formula:

$$\langle(\Delta Z)^2\rangle(T) = L_z^2\left[\frac{1}{6} - \frac{16}{\pi^4}\sum_{n=1}^{\infty}\frac{1}{(2n-1)^4}\exp\left(-\frac{(2n-1)^2\pi^2\, I_{D,Z}(T)}{L_z^2}\right)\right].$$

Substituting for $I_{D,Z}$ and $L_z = 110\,\mu$m,(so $L_z^2 = 12100\,\mu\mathrm{m}^2$), $D_0 = 1$ μm^2/ms, and $I_{D,Z}(T) = 1219.51\,\mu\mathrm{m}^2$, the argument of the exponential for the leading mode $n = 1$ is:

$$\frac{(2\times 1 - 1)^2\,\pi^2 \times 1219.51}{12100} = \frac{\pi^2 \times 1219.51}{12100} = 0.99426. \tag{60}$$

and

$$\frac{1}{(2n-1)^4}\exp(-0.99426) = 0.36992$$

Terms higher than $n = 1$ contribute less than 0.1% and can be neglected. Thus substituting into the MSD equation:

$$\begin{aligned}\langle(\Delta Z)^2\rangle(T) &= 12100\left[\frac{1}{6} - \frac{16}{\pi^4}\times 0.36993\right] \\ &= 1281.7\,\mu\mathrm{m}^2. \end{aligned} \tag{61}$$

with corresponding $D_{ZZ} = 1281.7/2(1000) = 0.6409$, far short of the case for an infinite domain eq. (57) where $\langle(\Delta Z)^2\rangle(T) \rightarrow 2I_{D,Z}$

For the no-strain case, $I_{D,Z}$ is simply $D_0T = 1000$ and substituting into the equation eq. (54) gives $\langle(\Delta Z)^2\rangle(T) = 1137.3\mu\mathrm{m}^2$.

2.3 Transformation to centred domain and propagator for particle release from centre

With the origin placed at the cell centre, the domain is $[-L/2,\, L/2]$ rather than $[0, L]$. Here we show that the MSD formula (53) is unchanged under this shift, and derive the simplified propagator for particles released from the centre. (The latter allows comparison with the finite volume solution for an impermeable cell undergoing deformation). Firstly with the coordinate translation:

$$\tilde{X} = X - \frac{L}{2}, \qquad \tilde{X}_0 = X_0 - \frac{L}{2}, \tag{62}$$

we have $\tilde{X}, \tilde{X}_0 \in [-L/2,\, L/2]$ when $X, X_0 \in [0, L]$. Since $X - X_0 = \tilde{X} - \tilde{X}_0$, the MSD integral (47) becomes:

$$\langle (X - X_0)^2 \rangle = \frac{1}{L} \int_{-L/2}^{L/2} \int_{-L/2}^{L/2} (\tilde{X} - \tilde{X}_0)^2\, G(\tilde{X}_0, \tilde{X}, t)\, d\tilde{X}\, d\tilde{X}_0, \tag{63}$$

which is identical in form to equation (47), just with $[0, L]$ replaced by $[-L/2,\, L/2]$.
Likewise, for the propagator in the new coordinates simply substitute $X = \tilde{X} + L/2$ and $X_0 = \tilde{X}_0 + L/2$ into equation (45):

$$G(\tilde{X}_0, \tilde{X}, t) = \frac{1}{L} + \frac{2}{L} \sum_{m=1}^{\infty} \cos\left(\frac{m\pi(\tilde{X}_0 + L/2)}{L}\right) \cos\left(\frac{m\pi(\tilde{X} + L/2)}{L}\right) \exp\left(-\frac{m^2\pi^2\, I_D(t)}{L^2}\right). \tag{64}$$

In Equation (63) we can evaluate the integrals over $\tilde{X}$ and $\tilde{X}_0$ by substituting $u = \tilde{X} + L/2$, $u_0 = \tilde{X}_0 + L/2$, which maps $[-L/2,\, L/2]$ back to $[0, L]$ and recovers the same integrals as before, so that

$$\langle (\tilde{X} - \tilde{X}_0)^2 \rangle = L^2 \left[\frac{1}{6} - \frac{16}{\pi^4} \sum_{n=1}^{\infty} \frac{1}{(2n-1)^4} \exp\left(-\frac{(2n-1)^2\pi^2\, I_D(t)}{L^2}\right) \right], \tag{65}$$

which is **identical** to equation (53). The averaged MSD is thus independent of origin placement.

Propagator for particles released from the cell centre. In this case all particles start with $\tilde{X}_0 = 0$, so in eq. eq. (64) we now have:

$$G(0, \tilde{X}, t) = \frac{1}{L} + \frac{2}{L} \sum_{m=1}^{\infty} \cos\left(\frac{m\pi}{2}\right) \cos\left(\frac{m\pi(\tilde{X} + L/2)}{L}\right) \exp\left(-\frac{m^2\pi^2\, I_D(t)}{L^2}\right). \tag{66}$$

Since $\cos(m\pi/2) = 0$ for all odd m, only even modes $m = 2n$ contribute; then for even $m = 2n$, $\cos(n\pi) = (-1)^n$, and:

$$\cos\left(\frac{2n\pi(\tilde{X} + L/2)}{L}\right) = \cos\left(\frac{2n\pi\tilde{X}}{L} + n\pi\right) = (-1)^n \cos\left(\frac{2n\pi\tilde{X}}{L}\right). \tag{67}$$

The $(-1)^n$ above now cancels with the $\cos(m\pi/2)$ in eq. (66), reducing the propagator to

$$G(0, \tilde{X}, t) = \frac{1}{L} + \frac{2}{L} \sum_{n=1}^{\infty} \cos\left(\frac{2n\pi\tilde{X}}{L}\right) \exp\left(-\frac{4n^2\pi^2\, I_D(t)}{L^2}\right). \tag{68}$$

All even modes contribute with equal positive weight, reflecting the symmetry of the problem about the release point $\tilde{X}_0 = 0$. The corresponding MSD for particles released from the centre is obtained by computing $\langle \tilde{X}^2 \rangle = \int_{-L/2}^{L/2} \tilde{X}^2\, G(0, \tilde{X}, t)\, d\tilde{X}$, which gives:

$$\langle \tilde{X}^2 \rangle \big|_{\tilde{X}_0 = 0} = \frac{L^2}{12} + \frac{L^2}{\pi^2} \sum_{n=1}^{\infty} \frac{(-1)^n}{n^2} \exp\left(-\frac{4n^2\pi^2\, I_D(t)}{L^2}\right). \tag{69}$$

This differs from the position-averaged formula (53) firstly in that the saturation value is $L^2/12$ rather than $L^2/6$ (since the maximum displacement from the origin is $L/2$ rather than L), and the series involves only even modes with alternating signs, decaying with argument $4n^2\pi^2 I_D/L^2$ rather than $(2n-1)^2\pi^2 I_D/L^2$.

3 Comparing Diffusion in the Extracellular and Intracellular Spaces

Figure 1 compares the principal components of the diffusion tensor based on the relevant coordinate MSD$/2T$ for the baseline geometry without strain, derived from the computational results. (Results shown from a single run with 10^6 particles, timestep $dt = 0.1$ ms; see below for convergence).

Unlike for the intracellular space, particles diffusing in the extracellular space are free to navigate the spaces between the cells. As permeability reduces, diffusion in both regions become more anisotropic, however whereas in the impermeable limit, diffusion inside the cells saturates, no such limit obtains for extracellular particles. Indeed the diffusion tensor components in the extracellular space appear constant, with the D_{xx} component lowest at $0.43D_0$, then D_{yy} at $0.72D_0$ and largely unrestricted $D_{zz} = 0.95D_0$.

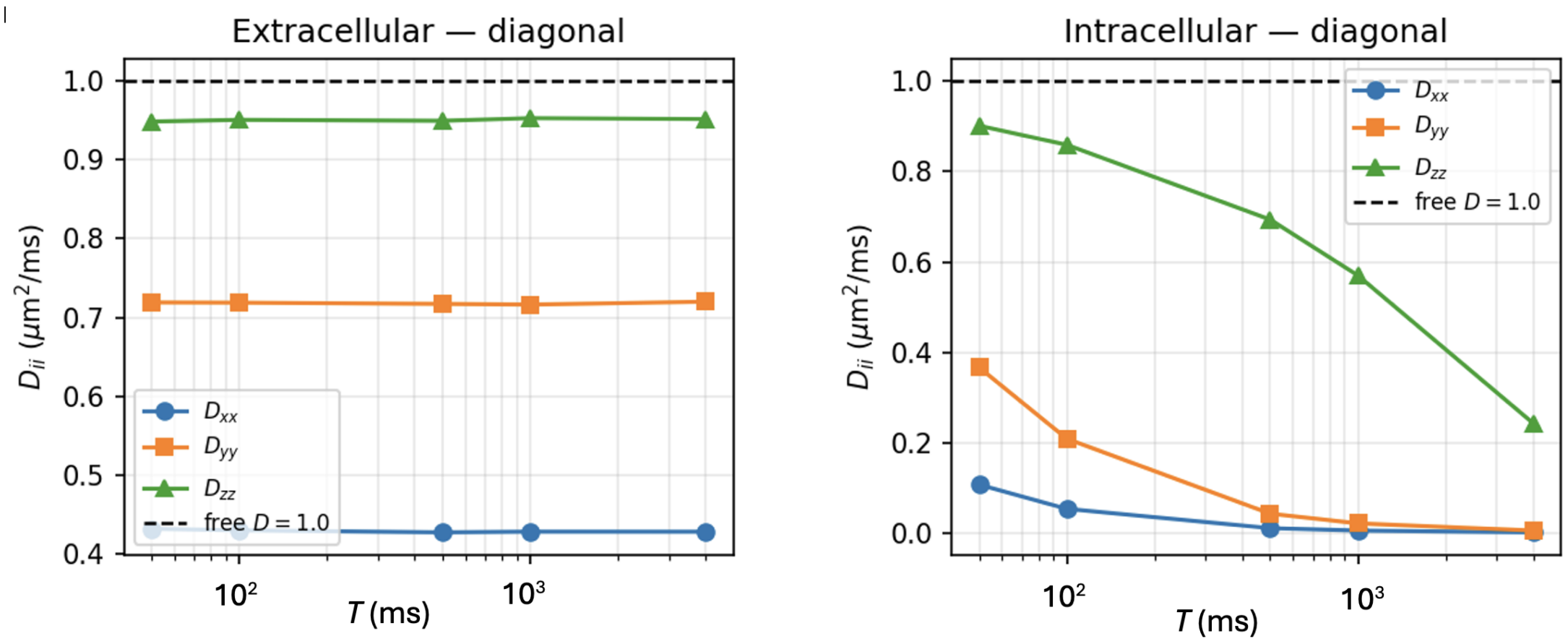

Figure 1: Principal diffusion tensor coefficients derived from MCRW computation. Left: for the extracellular space. Right: in the intracellular space

---

4 Computational Validation of MCRW Strain Model - General Case

The analytic methods described in the previous section are incapable of describing the complete geometry for arbitrary permeability. As an additional validation of the methodology for deformed media in MCRW algorithms, we implemented a finite volume (FV) method with deformable non-uniform rectilinear meshes. In this context, the evolution of concentration c in each control volume V is governed by:

$$\int_{\mathrm{V}} \frac{\partial c}{\partial t}\, dV + \int_{\partial \mathrm{V}} c\vec{v} \cdot \vec{n}\, dA = \int_{\partial \mathrm{V}} (D\nabla c) \cdot \vec{n}\, dA \tag{70}$$

where the right-hand side represents diffusive flux across faces (with diffusion coefficient D), and $\vec{n}$ the face normal vector. The second term on the left-hand side comes from the Reynolds Transport Theorem and is the advective transport due to mesh deformation (with face velocity v). The mesh vertices are updated according to the prescribed strain components $\epsilon_i(t)$ along each direction i, ensuring volume preservation. For numerical stability, the explicit time-stepping scheme must satisfy both the diffusive condition $\Delta t \leq \frac{(\Delta x)^2}{6D}$ and the CFL condition $\Delta t \leq \frac{\Delta x}{\max |v|}$, where Δx is the smallest grid spacing, and v is the mesh velocity.

A simplified domain featuring semi-permeable membranes was utilized to validate our diffusion-strain model (see Figure 2). We examined membrane permeabilities (κ) of 0, 0.025, 0.1, and 1.0 μm/ms, alongside a free-diffusion scenario where $\kappa = \infty$. A non-uniform mesh, more closely spaced near the cell membranes, was employed to accurately capture concentration gradients.

A brief linear compression was applied along the cell's longitudinal axis (z-axis), achieving a maximum strain (ε_{zz}) max of -40% at 125 millisecond, before returning to baseline by 250 millisecond. The deformation gradient is thus identical to that of the compression cycle 1, but for a much greater maximum strain and one quarter of the cycle time.

For validation purposes, both Finite Volume (FV) and MCRW methods are compared. An initial concentration ($c_0 = 1$) is considered at the domain's center, diffusing over $t = 250$ ms. In the FV method, this entailed

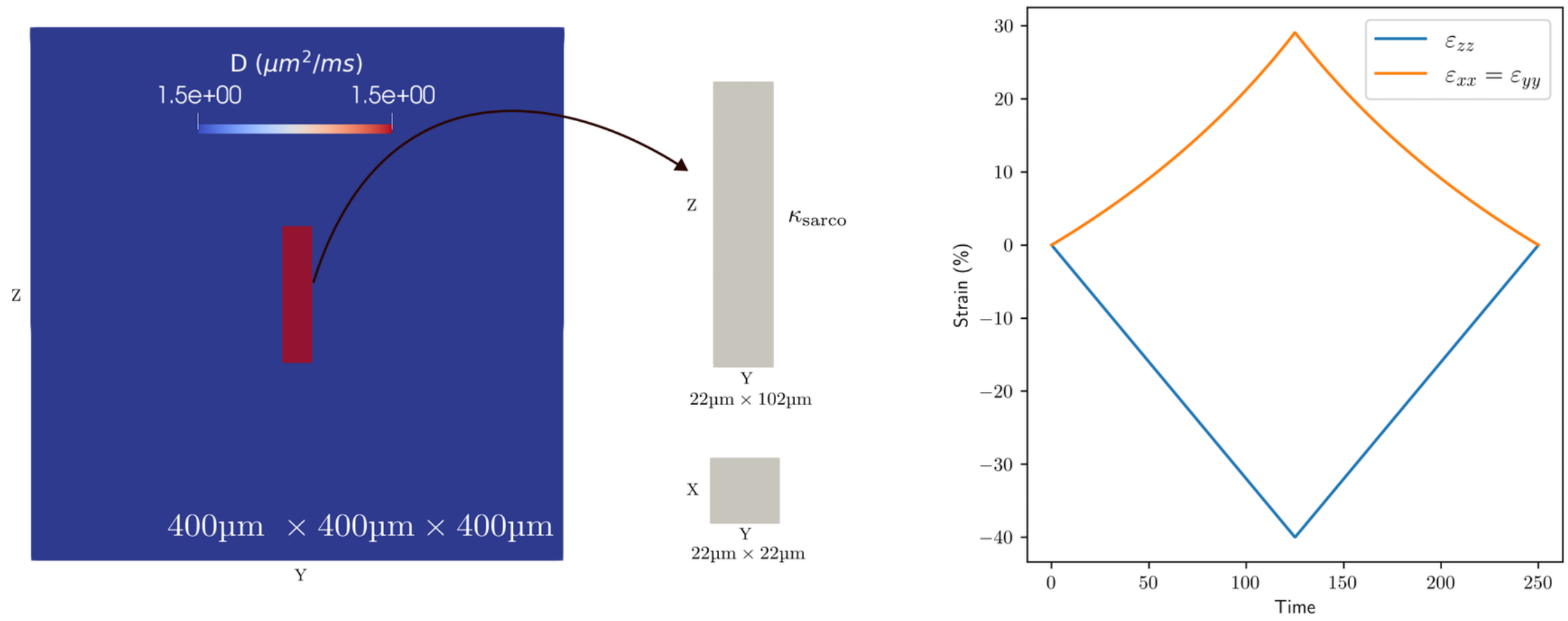

Figure 2: 2D representation of the 3D simplified domain, which models a single cardiomyocyte (red) as a 3D prism with dimensions $22 \times 22 \times 102\,\mu\text{m}^3$, within a $400 \times 400 \times 400\,\mu\text{m}^3$ domain, considered for validating the strain methodology in the MCRW model. The strain applied is brief and intense, with a 40% compression along the long axis of the cardiomyocyte and an approximate 28% expansion in other directions to preserve volume. These strain values exceed those typically observed in-vivo under physiological conditions, providing a challenging validation of the strain affected MCRW algorithm.

---

assigning concentration $c = 1$ to the central finite volume element, while in the MCRW method, particles were initially seeded at the domain's center.

In Supplementary Figure 3, the analytic solution for infinite medium (infinite permeability) is compared with the results using FV and MCRW methods. As is derived in earlier sections, the apparent diffusion in the z direction is increased by the factor $1/(1 + \varepsilon_{max})$, in this case $1/0.6 = 1.67$, whilst that in the x, y directions is reduced to $(2 + \varepsilon_{max})/2 = 0.8$.

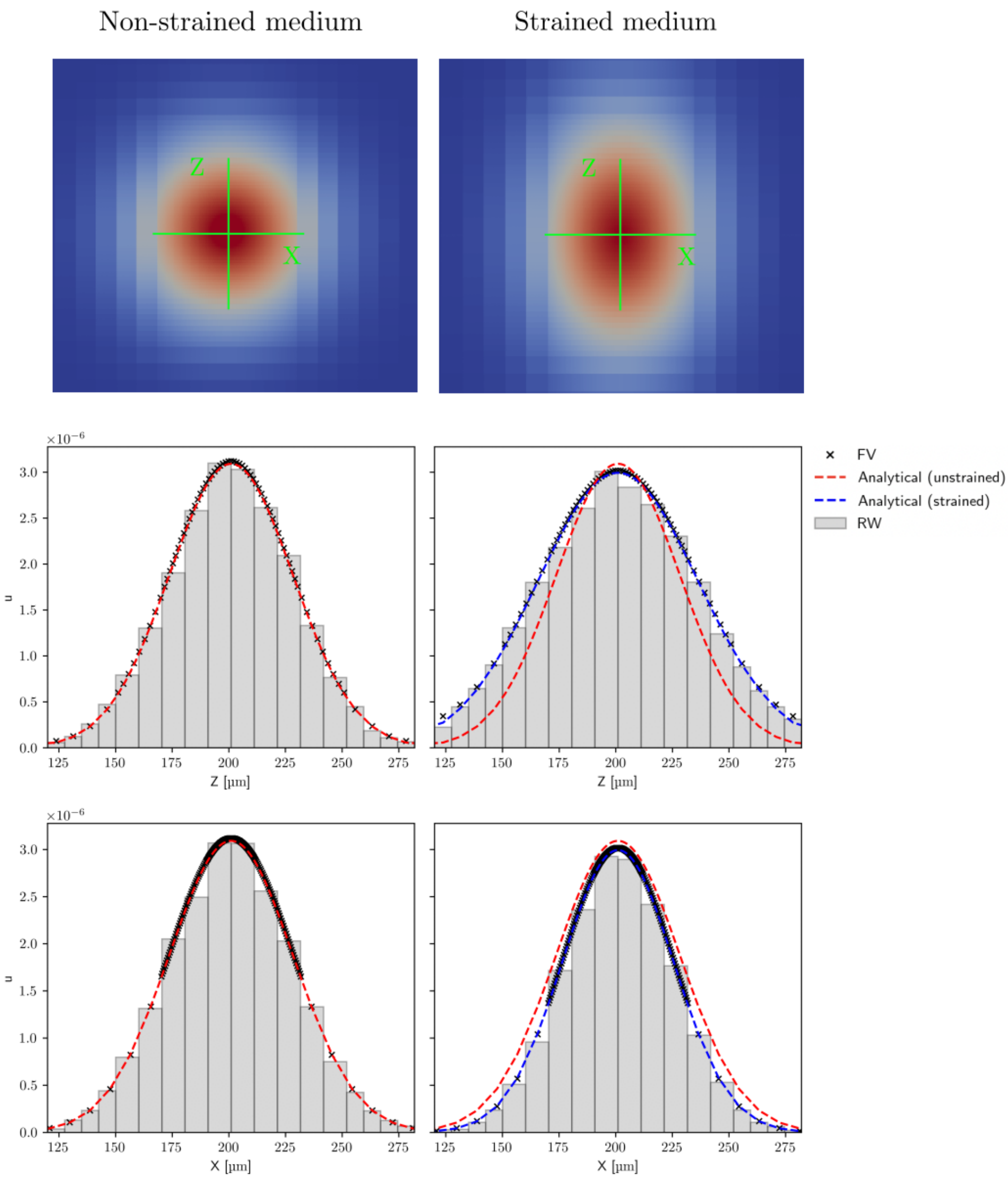

Figure 3: Free-diffusion results comparing the non-strained and strained media illustrating the particle concentration along the X and Z directions, presented as 2D histograms (upper panes) and 1D histograms of walker density in Z (middle row) and X (lower row). It is observed that compression increases the effective diffusion, while expansion reduces it. The random walk simulation results are validated against the analytical solution and the finite volume (FV) simulation results, demonstrating close correspondence between all methods.

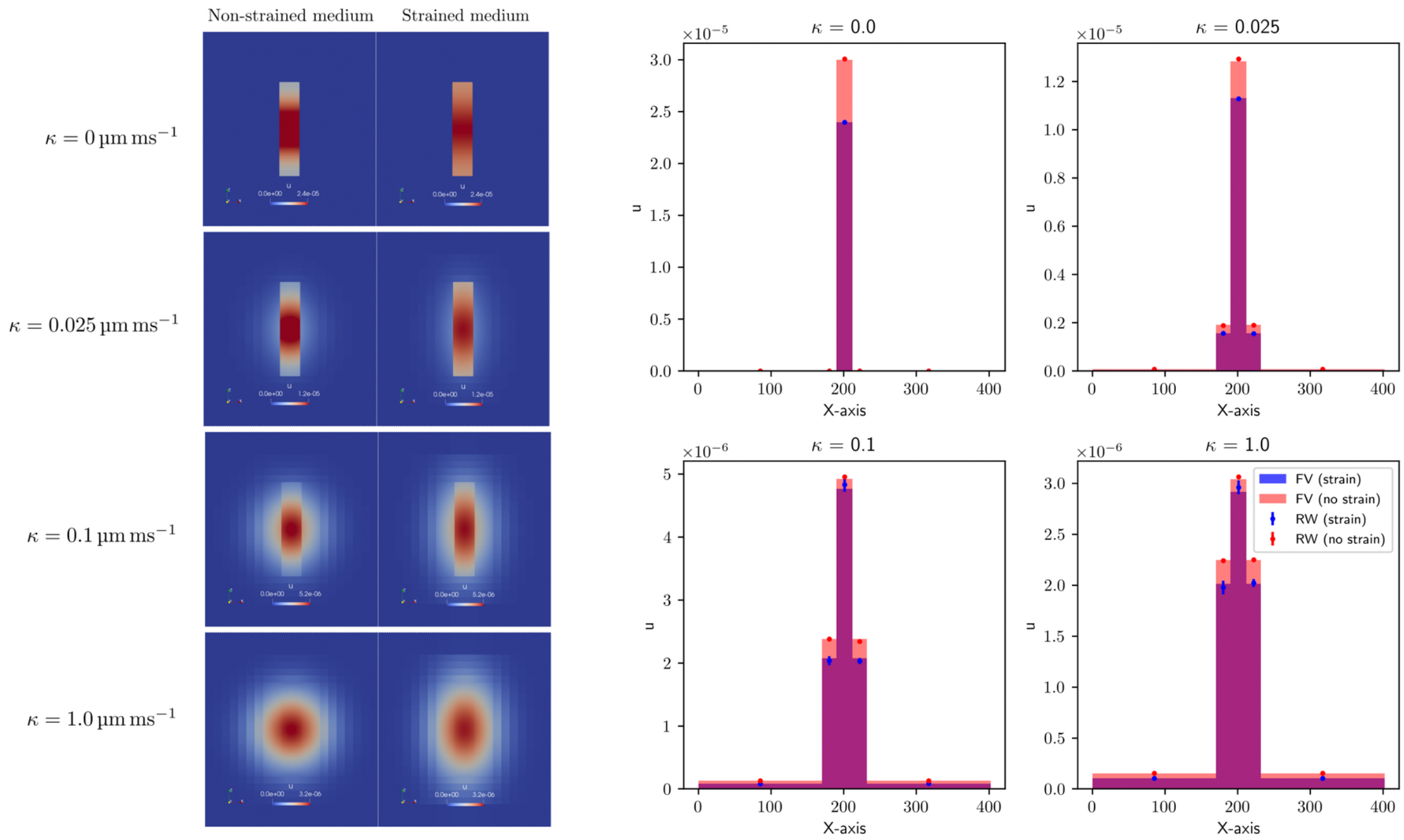

Figure 4: Comparison of finite volume (FV) and MCRW simulation results for various permeability values. *Left*: 2D $x - z$ plane slices (2D histograms) of the single-cell domain showing the FV simulation results for various permeability values, $\kappa = 0; 0.025\,\mu\text{m}\,\text{ms}^{-1}; 0.1\,\mu\text{m}\,\text{ms}^{-1}; 1\,\mu\text{m}\,\text{ms}^{-1}$, in both non-strained and strained media, following the brief intense compressive strain function illustrated in fig. 2.
Right: Histograms displaying the average concentrations along the x-axis for the four different permeability values, comparing the FV method with the Random Walk simulations.
It is observed that compression along the long axis of the cardiomyocyte enhances diffusion, while expansion in both transverse axes decreases overall diffusivity. In the top left of the four histograms (impermeable cell), the concentration is reduced to approximately 80% of that with no strain on the central slice, since strain has increased diffusivity in z, hence displacing more particles away from their central origin. As permeability increases, particl are progressively less confined and spread further in all directions. Note the close agreement between repeated (see standard deviation bars) random walk simulations and the FV computations for the mean concentration in the histogram plots across the range of permeability values.

In figure 4 we validate the model for different permeability values comparing both finite volume and MCRW methods across multiple compartments along the x and z axes. The MCRW simulations apportion particles to a coarser mesh with bin sizes aligned to the compartment structure: histograms plot the binned concentration at the mid-height in z of the model cardiomyocyte with fixed bin height in z ($1/11$ of the $z-$extent) and variable width bin in x with the central bin covering the total $x-$extent of 22 μm.

4.1 Comparison with theory

We can compare the concentration in the thick xy planar slices through the centre of the cell for the impermeable case, fig. 3 right panel. The histograms are derived from particle concentrations in a planar slice centred at the mid-height in z of the cell, where $2\delta = L_z/11$. Computationally this corresponds to binning all particles with $z \in \left[-\frac{L_Z}{22}, \frac{L_Z}{22}\right]$

This corresponds to the probability of particles being found in the slice. From the theory developed earlier, since the 3D concentration factorises and the x and y integrals over the full domain equal 1:

$$P_{\text{slice}}(T) = \underbrace{\int_{-L_X/2}^{L_X/2} G(0,x,T)\,dx}_{=1} \cdot \underbrace{\int_{-L_Y/2}^{L_Y/2} G(0,y,T)\,dy}_{=1} \cdot \int_{-\delta}^{+\delta} G(0,z,T)\,dz = \int_{-\delta}^{+\delta} G(0,z,T)\,dz. \tag{71}$$

Substituting equation (68) and integrating term by term:

$$P_{\text{slice}}(T) = \frac{1}{L_Z}\int_{-\delta}^{+\delta}\left[1 + 2\sum_{n=1}^{\infty}\cos\left(\frac{2n\pi z}{L_Z}\right)e^{-4n^2\pi^2 I_{D,Z}/L_Z^2}\right]dz$$

$$= \frac{2\delta}{L_Z} + \frac{2}{L_Z}\sum_{n=1}^{\infty}e^{-4n^2\pi^2 I_{D,Z}/L_Z^2}\underbrace{\int_{-\delta}^{+\delta}\cos\left(\frac{2n\pi z}{L_Z}\right)dz}_{=(L_Z/n\pi)\sin(2n\pi\delta/L_Z)}. \tag{72}$$

Therefore:

$$P_{\text{slice}}(T) = \frac{2\delta}{L_Z} + \frac{2}{\pi}\sum_{n=1}^{\infty}\frac{1}{n}\sin\left(\frac{2n\pi\delta}{L_Z}\right)\exp\left(-\frac{4n^2\pi^2\, I_{D,Z}(T)}{L_Z^2}\right). \tag{73}$$

With $\delta = L_Z/22$, the geometric prefactor and sine arguments are:

$$\frac{2\delta}{L_Z} = \frac{1}{11} = 0.090909, \qquad \frac{2n\pi\delta}{L_Z} = \frac{2n\pi\cdot L_Z/22}{L_Z} = \frac{n\pi}{11} = 0.28560\,n \text{ rad}. \tag{74}$$

Numerical values of $\sin(n\pi/11)$ are: $n = 1,\ \sin(n\pi/11) = 0.2817$; $n = 2,\ \sin(n\pi/11) = 0.5469$.

Strained case: Evaluating $I_{D,Z}(T)$ yields 416.67 μm^2. Then for $n = 1$ the contribution to the sum is 0.0580; for $n = 2$, we add 0.0049 to this yielding 0.05846. Higher order terms are negligible. Hence

$$P_{\text{slice}}^{\text{strain}}(T) \approx \frac{1}{11} + \frac{2}{\pi}\times 0.05846 = 0.09091 + 0.03722 = 0.12813. \tag{75}$$

Unstrained case: Similarly, for the unstrained case, $I_{D,Z}(T) = 250\ \mu\text{m}^2$ and the sum $= 0.10908 + 0.006137 + 0.0000486 = 0.11527$.

$$P_{\text{slice}}^{\text{no strain}}(T) \approx \frac{1}{11} + \frac{2}{\pi}\times 0.11527 = 0.090909 + 0.073396 = 0.16430. \tag{76}$$

Concentration Ratio

$$\frac{P_{\text{slice}}^{\text{strain}}(T)}{P_{\text{slice}}^{\text{no strain}}(T)} = \frac{0.12813}{0.16430} = 0.7799, \tag{77}$$

which agrees well with the ratio of strained to unstrained values in the top left histogram plot, where the concentration ratio is ~ 0.79, showing good agreement between FV and MCRW under strain.

5 MCRW Convergence - effect of reduced frequency of strain updates

The convergence studies have been conducted considering geometry C1 with linear compression (cycle 1). For diffusion convergence analysis (without strain), we examined the most restrictive scenario where barriers are completely impermeable ($\kappa = 0\,\mu\text{m}\,\text{ms}^{-1}$). Impermeability presents the most challenging convergence for diffusion as particles experience maximum restriction in their movement. By ensuring convergence in this limiting case, we establish confidence in the algorithm's performance across all permeability values.

For the strain time step convergence analysis, we selected $\kappa = 1\,\mu\text{m}\,\text{ms}^{-1}$ to investigate the relationship between diffusion time steps (Δt_D) and strain time steps (Δt_ε). This analysis is particularly significant as it determines whether the two temporal scales must be synchronized or if they can operate at different rates without compromising diffusion tensor accuracy. By allowing Δt_ε to be larger than Δt_D, we effectively update the geometry less frequently, creating a step-wise approximation of the continuous strain function. This approach potentially offers substantial computational savings if the diffusion tensor parameters remain stable despite the coarser strain time discretization.

5.1 Diffusion (without strain)

To validate the convergence of the MCRW algorithm without deformation with respect to the number of particles N_p and time-steps N_t, we performed a statistical analysis using the most restrictive case of completely impermeable boundaries $\kappa = 0\,\mu\text{m}\,\text{ms}^{-1}$. First, we conducted a base simulation with $N_p = 10^6$ particles over 1000 ms using $N_t = 10^4$ time steps, at which temporal value simulations were well converged.

Then, to analyze the effect of particle count on convergence, we employed a bootstrapping approach: for each smaller particle count (N_p ranging from 10^3 to 10^6), we randomly sampled particles from the base simulation population 50 times to assess statistical variability. The convergence analysis focused on the eigenvalues $\lambda_{1,2,3}$ of the diffusion tensor computed from these bootstrapped samples. The statistical distribution of these eigenvalues across the 50 bootstrap iterations provides a measure of the stability and accuracy. Figure 5

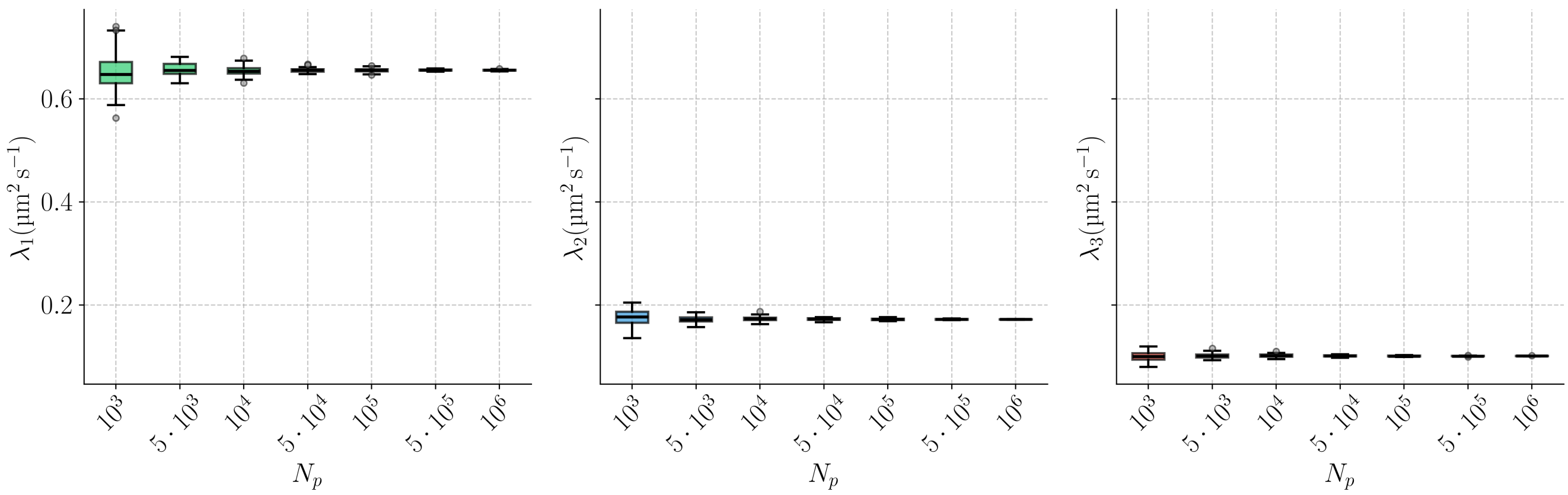

Figure 5: Convergence analysis of the MCRW algorithm as a function of particle count (N_p) for a fixed number of time steps $N_t = 10^4$. Box plots showing the statistical distribution of diffusion tensor eigenvalues λ_1, λ_2, and λ_3 across 50 bootstrap iterations for distinct number of particles N_p ranging from 10^3 to 10^6. The results demonstrate that $N_p = 5 \cdot 10^5$ provides sufficient accuracy while maintaining computational efficiency, with variance reduction becoming minimal beyond this threshold.

shows the reduction in variance and stabilization of the median λ values as N_p increases. Using $N_p = 5 \cdot 10^5$ and $N_t = 10^4$ is sufficient to accurately and reliable compute diffusion tensor parameters while maintaining computational efficiency.

5.2 Diffusion with strain convergence

Simulations using $N_p = 5 \cdot 10^5$ particles were performed with time steps of 0.1 ms ($N_t = 10^4$ timesteps). Compared with the theoretical result for the impermeable cell only, this choice of parameters yielded relative errors in the computed diffusion tensor components <0.1%, 2.6%, 3.5% for respectively D_{zz}, D_{yy}, D_{xx}. However most of the contributions to D_{yy}, D_{xx} come from the extracellular spaces, so those values do not reflect the overall error. For the entire domain there is no analytic solution except for infinite permeability where computations and theory match. Based on computations at higher resolution, we are at least confident the error in reported diffusion coefficients is everywhere below 0.5% with 10^5 particles and dt of 0.1 ms. This threshold is considered adequate, given the many assumptions in the basic model.

To determine the necessary frequency of strain updates, we ran simulations with $N_p = 5 \cdot 10^5$ particles and $N_t = 10^4$ time steps with different strain time steps, having different ratios $\Delta t_\epsilon / \Delta t_D$ between diffusion and strain steps during simulations of total time $T = 1000\,\text{ms}$. Note that $\Delta t_\epsilon / \Delta t_D = 1$ means having the same diffusion and strain time step of $\Delta t_\epsilon = \Delta t_D = 0.1\,\text{ms}$ over the 1000 ms simulation.

We consider different strain steps of 0.1 ms, 1 ms, 10 ms, 50 ms, 100 ms, 250 ms, 500 ms and 1000 ms, where a strain step of 1000 ms effectively means no strain is applied. Figure 6 illustrates the mean and standard deviation of 50 bootstrap samples for each strain time step. The convergence strain analysis shows that using $\Delta t_\epsilon = 100\,\text{ms}$ converges to similar parameters to using the smallest strain steps. This result shows that for the period (1000 ms) of the strain cycles considered, the geometry appears quasi-steady relative to the Brownian motion of the particles.

Conclusion: the fact that strain steps can be many orders larger than diffusion steps for the moderate strain rates considered not only allows considerable computational savings, but indicates that models of permeability derived for the unstrained case carry over without modification to cases with strain.

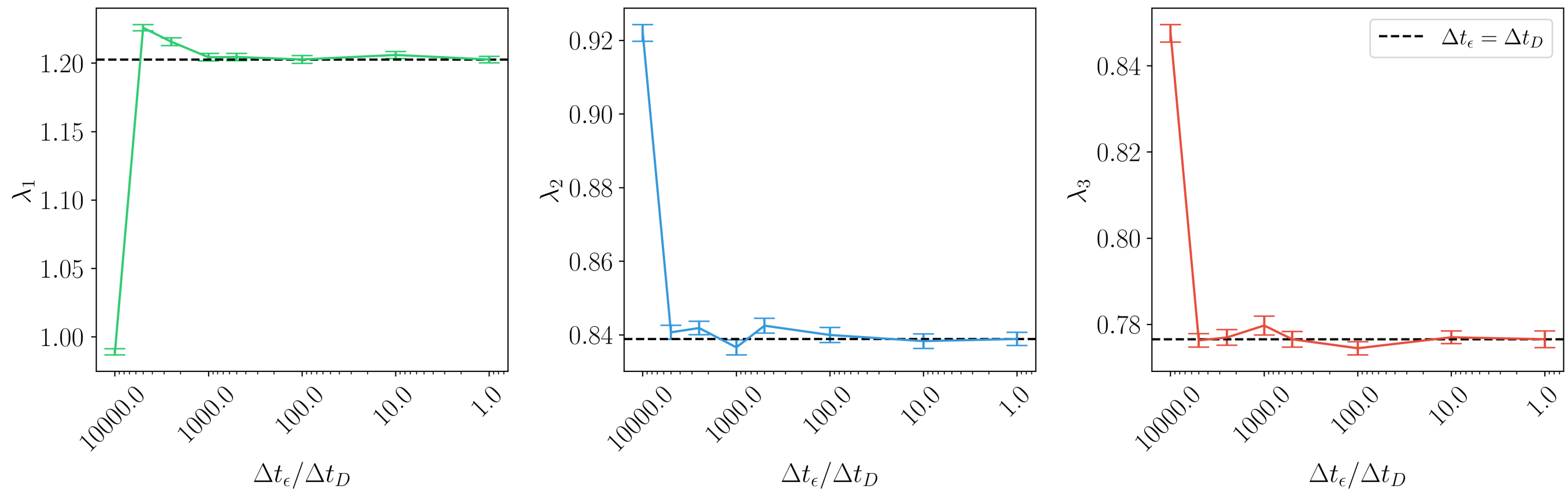

Figure 6: Convergence analysis for strain time step resolution. Mean and standard deviation of diffusion tensor eigenvalues λ_1, λ_2, and λ_3 from 50 bootstrap samples at different strain-to-diffusion time step ratios ($\Delta t_\varepsilon/\Delta t_D$). The analysis indicates that $\Delta t_\varepsilon = 100\,\text{ms}$ (ratio = 1000) provides an optimal balance between accuracy and computational efficiency.

6 Simple MATLAB scripts for diffusion with strain in homogeneous media

In this section we provide sample Matlab scripts to determine the effects of strain on diffusion for cycle 1 (C1 compressed to C2 and expanded back to C1) and for the extreme test case where initial geometry C1 is compressed by 40% and re-expanded to its original size. Scripts are simplified for clarity rather than optimal efficiency.

Either script is easily adapted to represent any arbitrary strain profile. However in the first script we apply a constant incremental compression. This does not exactly reproduces the linear progressive strain but allows a simple comparison with the analysis presented and the difference between the effect of the exact and approximate strain profiles is minimal. The second script does apply a linear progressive strain since the approximation is not so accurate for a compression by 40%.

6.1 Script 1: approximate cycle 1: C1 –> C2 –> C1.

```
% Effect of strain on diffusion
% Case C1: linearly increasing compression by 18% from 0 to 500 ms
% followed by reversion to initial state - approximated as constant
% multiplicative strain. For exact linear see other script.
%
clear; close all;
npoints  = 100000;
nbatches = 12;
%Initial assumed isotropic diffusion
D  = [1, 0  0; 0,  1,  0; 0,  0, 1]
T  = 1000;
dt = 0.01;

ntsteps = fix(T/dt)
Dscale(1) = sqrt(2*dt*D(1,1));
Dscale(2) = sqrt(2*dt*D(2,2));
Dscale(3) = sqrt(2*dt*D(3,3));
xn = ones(nbatches, npoints);
yn = ones(nbatches, npoints);
zn = ones(nbatches, npoints);
% set  nominal total strain = strain rate epsilon_dot x T
e1_total = -0.18
e2_total = 1/(1+e1_total) - 1
```

```
e1dot_dt = (log(1+e1_total))/(ntsteps/2)
e2dot_dt = (log(1+e2_total))/(ntsteps/2)

tic
parfor i = 1:nbatches
%for i = 1:nbatches
x = zeros(1,npoints);
y = zeros(1,npoints);
z = zeros(1,npoints);
t = 0;
for n = 1:ntsteps/2
t = t + dt;
dx =  Dscale(1)*sign(rand(1, npoints) - 0.5);
x = x + dx;
x = (1+e1dot_dt)*x;
dy = Dscale(2)*sign(rand(1, npoints) - 0.5);
y  = y + dy;
y =  y/(sqrt(1+e1dot_dt));
dz = Dscale(3)*sign(rand(1, npoints) - 0.5) ;
z  = z + dz ;
z =  z/(sqrt(1+e1dot_dt));
end

for n = ntsteps/2+1:ntsteps
t = t + dt;
dx =  Dscale(1)*sign(rand(1, npoints) - 0.5);
x = x + dx;
x = (1+e2dot_dt)*x;
dy = Dscale(2)*sign(rand(1, npoints) - 0.5);
y  = y + dy;
y =  y/(sqrt(1+e2dot_dt));
dz = Dscale(3)*sign(rand(1, npoints) - 0.5) ;
z  = z + dz ;
z =  z/(sqrt(1+e2dot_dt));
end
xn(i, :) = x;
yn(i, :) = y;
zn(i, :) = z;
end
toc
xp = reshape(xn, [nbatches * npoints, 1]);
yp = reshape(yn, [nbatches * npoints, 1]);
zp = reshape(zn, [nbatches * npoints, 1]);

% calculate covariance matrix of displacement for each case
disp = [xp(:), yp(:), zp(:)];
cov_disp = cov(disp);
D_calc = cov_disp/(2*(T));
% print D_calc to screen
D_calc
% calculate theoretical apparent diffusion tensor
fprintf('Theoretical D_app \n')
aT = -2*log(1+e1_total)
D_xx_app = 2*(1-exp(-aT/2))/aT
D_yy_app = D_xx_app
D_zz_app = (exp(aT)-1)/aT
D_app = [D_xx_app, 0, 0; 0, D_yy_app, 0; 0, 0, D_zz_app]

fprintf('\n eigenvalues of calculated strained diffusion =\n')
fprintf('%.2f %.2f %.2f\n', eig(D_calc))
fprintf('\n eigenvalues of theoretical strained diffusion =\n')
fprintf('%.2f %.2f %.2f\n', eig(D_app))
```

```
scatter3(xp, yp, zp, 'k.')
```

6.2 Script 2: exact linear strain cycle 1: C1 –> C2 –> C1.

```
clear; close all;
npoints  = 200000;
nbatches = 12;
%Initial assumed isotropic diffusion
D  = [1, 0  0; 0,  1,  0; 0,  0, 1]
T  = 250;
dt = 0.05;
ntsteps = fix(T/dt)
Dscale(1) = sqrt(2*dt*D(1,1));
Dscale(2) = sqrt(2*dt*D(2,2));
Dscale(3) = sqrt(2*dt*D(3,3));
xn = ones(nbatches, npoints);
yn = ones(nbatches, npoints);
zn = ones(nbatches, npoints);
% set  nominal total strain
e1_total = -0.4               % first half cycle (0 - T/2) is compression
e2_total = 1/(1+e1_total) - 1
e1dot_dt = e1_total/(ntsteps/2);
e2dot_dt = e2_total/(ntsteps/2);

tic   % start execution timer
parfor i = 1:nbatches
% if not using parallel library use: for i = 1:nbatches
x = zeros(1,npoints);
y = zeros(1,npoints);
z = zeros(1,npoints);
t = 0;

for n = 1:ntsteps/2   % first half cycle
Fnx = 1 + (e1dot_dt/(1 + n*e1dot_dt));
a = 1 + n*e1dot_dt;
Fny =  1/(sqrt(1 + e1dot_dt/a));
Fnz = 1/(sqrt(1 + e1dot_dt/a));
t = t + dt;
dx = Dscale(1)*sign(rand(1, npoints) - 0.5);
dy = Dscale(2)*sign(rand(1, npoints) - 0.5);
dz = Dscale(3)*sign(rand(1, npoints) - 0.5) ;
% advance particle via diffusive step
x = x + dx;
y  = y + dy;
z  = z + dz ;
% now apply strain in x
x = Fnx*x;
% next strain in y, z
y = Fny*y;
z = Fnz*z;
end

for n = 1:ntsteps/2   % second half cycle
Fnx = 1 + (e2dot_dt/(1 + n*e2dot_dt));
a = 1 + n*e2dot_dt;
Fny =  1/(sqrt(1 + e2dot_dt/a));
Fnz = 1/(sqrt(1 + e2dot_dt/a));
t = t + dt;
dx =  Dscale(1)*sign(rand(1, npoints) - 0.5);
dy = Dscale(2)*sign(rand(1, npoints) - 0.5);
dz = Dscale(3)*sign(rand(1, npoints) - 0.5) ;
% advance particle via diffusive step
```

```
x = x + dx;
y  = y + dy;
z  = z + dz ;
x = Fnx*x;
y = Fny*y;
z = Fnz*z;
end
xn(i, :) = x;
yn(i, :) = y;
zn(i, :) = z;
end
toc % stop execution timer
xp = reshape(xn, [nbatches * npoints, 1]);
yp = reshape(yn, [nbatches * npoints, 1]);
zp = reshape(zn, [nbatches * npoints, 1]);

% calculate covariance matrix of displacement for each case
disp = [xp(:), yp(:), zp(:)];
cov_disp = cov(disp);
D_calc = cov_disp/(2*(T));
% print D_calc to screen
D_calc
% Write out the theoretical apparent diffusion for this case
fprintf('Theoretical D_apparent \n')
D_app = [D(1,1)*(1/(1 + e1_total)), 0  , 0; 0, ...
D(2,2)*(1 + e1_total/2), 0; 0,  0, D(3,3)*(1 + e1_total/2)]

fprintf('\n eigenvalues of calculated strained diffusion =\n')
fprintf('%.2f %.2f %.2f\n', eig(D_calc))
fprintf('\n eigenvalues of theoretical strained diffusion =\n')
fprintf('%.2f %.2f %.2f\n', eig(D_app))
%opfile=input('enter filename for ascii o/p : strained diffusion\n','s' );
%opfile
%save(opfile,'xp','yp','zp','-ascii');
scatter3(xp, yp, zp, 'k.')
```

References

[1] Reese, T. G. et al. (1995)Imaging myocardial fiber architecture in vivo with magnetic resonance. Magn. Reson. Medicine 34, DOI: 10.1002/mrm.1910340603.

[2] Reese, Timothy G. Van Wedeen, J. and Weisskoff, Robert M., (1996) Measuring diffusion in the presence of material strain, Journal of Magnetic Resonance - Series B, 3, 112, 10.1006/jmrb.1996.0139, issn = 10641866

[3] Stejskal, E. O. & Tanner, J. E. (1965) Spin diffusion measurements: Spin echoes in the presence of a time-dependent field gradient. The J. Chem. Phys. 42, DOI: 10.1063/1.1695690 (1965).

[4] Crank, J. The mathematics of diffusion. Oxford University Press ISBN 13:9780198534112 (1979).
